# Emplacement of the Franklin large igneous province and initiation of the Sturtian Snowball Earth

**DOI:** 10.1126/sciadv.adc9430

**Published:** 2022-11-23

**Authors:** Judy P. Pu, Francis A. Macdonald, Mark D. Schmitz, Robert H. Rainbird, Wouter Bleeker, Barra A. Peak, Rebecca M. Flowers, Paul F. Hoffman, Matthew Rioux, Michael A. Hamilton

**Affiliations:** ^1^ Department of Earth and Planetary Sciences, Harvard University, Cambridge, MA, USA.; ^2^ Department of Earth Science, University of California, Santa Barbara, Santa Barbara, CA, USA.; ^3^ Department of Geosciences, Boise State University, Boise, ID, USA.; ^4^ Geological Survey of Canada, Ottawa, Ontario, Canada.; ^5^ Department of Geological Sciences, University of Colorado, Boulder, CO, USA.; ^6^ School of Earth and Ocean Sciences, University of Victoria, Victoria, British Columbia, Canada.; ^7^ Department of Earth Sciences, University of Toronto, Toronto, Ontario, Canada.

## Abstract

During the Cryogenian (720 to 635 Ma ago) Snowball Earth glaciations, ice extended to sea level near the equator. The cause of this catastrophic failure of Earth’s thermostat has been unclear, but previous geochronology has suggested a rough coincidence of glacial onset with one of the largest magmatic episodes in the geological record, the Franklin large igneous province. U-Pb geochronology on zircon and baddeleyite from sills associated with the paleo-equatorial Franklin large igneous province in Arctic Canada record rapid emplacement between 719.86 ± 0.21 and 718.61 ± 0.30 Ma ago, 0.9 to 1.6 Ma before the onset of widespread glaciation. Geologic observations and (U-Th)/He dates on Franklin sills are compatible with major post–Franklin exhumation, possibly due to development of mafic volcanic highlands on windward equatorial Laurentia and increased global weatherability. After a transient magmatic CO_2_ flux, long-term carbon sequestration associated with increased weatherability could have nudged Earth over the threshold for runaway ice-albedo feedback.

## Teaser

Volcanic highlands in the tropics increased global weatherability ~0.9–1.6 Ma before onset of Cryogenian Snowball Earth.

## INTRODUCTION

Sedimentological, paleomagnetic, and geochronological data have established the presence of ice at low latitudes during the Cryogenian period (720 to 635 Ma ago), providing evidence for two global glaciations, the Sturtian (~717 to 659 Ma ago) and Marinoan (>639 to 635 Ma ago) (*1*). The Snowball Earth episodes are the most extreme climate changes in Earth’s history, and, yet, the triggers for their initiation remain unclear and debated. Studies of the emplacement of large igneous provinces (LIPs) have demonstrated correlations with environmental perturbations (*2*, *3*), and geochronological constraints on the older of the two episodes, the Sturtian Snowball Earth, have highlighted a possible correlation in timing of onset with the emplacement of the Franklin LIP [e.g., (*4*, *5*)].

Previous work has attributed the global cooling that led to the Sturtian glaciation to LIP emplacement, because of either drawdown of CO_2_ by silicate weathering for millions of years following eruption of the Franklin LIP (*4*, *6*–*8*) or the immediate effects from the radiative forcing of sulfur aerosol emissions (*5*). Several paleomagnetic studies have demonstrated that the Franklin LIP was emplaced at tropical latitudes [e.g., (*9*, *10*)], which is critical for both hypotheses as the climate impact of changes in weatherability and albedo are both strongly latitude dependent (*5*, *6*). Past U-Pb geochronology studies have also produced emplacement ages for the Franklin LIP ranging from 750 to 710 Ma (*4*, *10*–*13*), which roughly coincide with constraints on the establishment of steady-state ice margins and the onset of Sturtian glaciation between 717.4 ± 0.2 and 716.9 ± 0.4 Ma (**14**), but the published uncertainties are too large to determine whether there is a causal link between LIP emplacement and the onset of the Sturtian glaciation or differentiate between the proposed climate cooling mechanisms.

In the past several years, geochronology studies have constrained the duration and tempo of LIP emplacement, showing that >75% of total volume is emplaced in pulses of <5 Ma, with almost all high-precision studies showing emplacement in <1 Ma (*3*, *15*). On the basis of the established rates for LIP emplacement, the actual duration of emplacement for the Franklin LIP could be only a small portion of the current range in dates, highlighting the need for higher precision and accuracy in dating.

Obstacles to the accurate and precise dating of Proterozoic LIPs have included the lack of felsic, zircon-rich rocks and the extent of radiation damage in Proterozoic zircon grains, which often leads to pervasive Pb loss. In this study, we used U-Pb isotope dilution–thermal ionization mass spectrometry (ID-TIMS) on zircon and baddeleyite extracted from intermediate to felsic differentiates of the Franklin LIP from locations across Arctic Canada. Whole-rock geochemistry was used to place precisely dated samples in the context of an emplacement model to clarify the relationship between the Franklin LIP and the onset of glaciation. To minimize the impact of Pb loss, we used the chemical abrasion method [CA-ID-TIMS; (*16*)], in most cases dating minute, remnant fragments from aggressive dissolution of uranium-rich zircon crystals. Apatite and zircon (U-Th)/He thermochronology dates were also obtained and combined with geologic observations to evaluate the timing and magnitude of postemplacement exhumation.

### Geologic setting

**Fig. 1.**
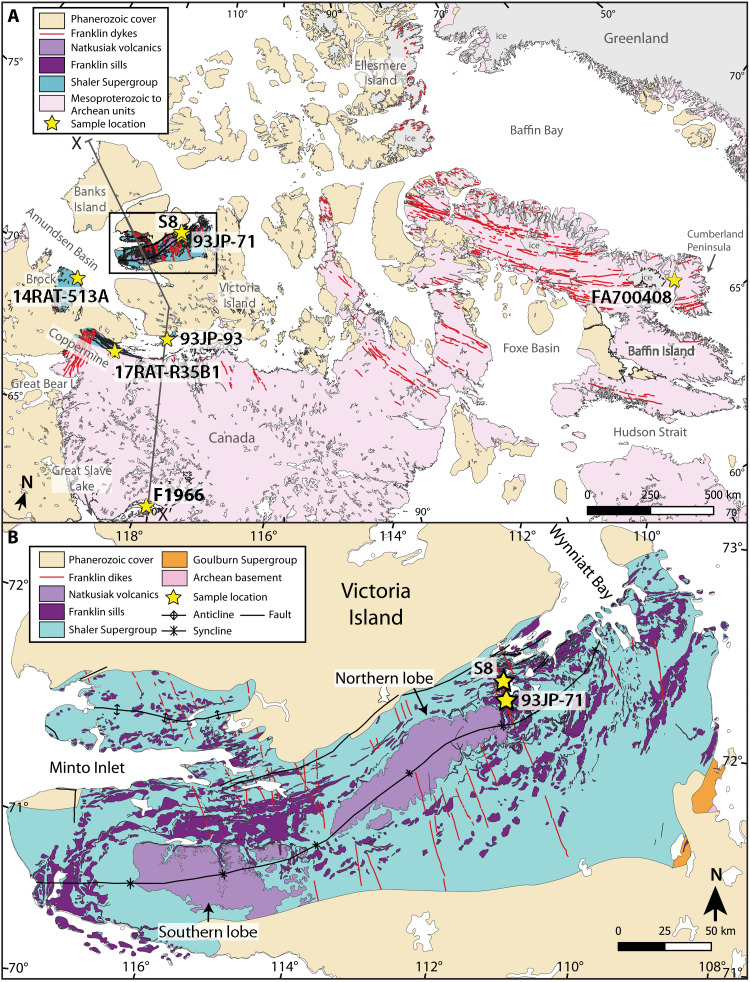
Franklin LIP and sample map. Maps were modified from QGIS shape files from (*72*–*74*). A Canada polar stereographic projection based on WGS 1984 was used [European Petroleum Survey Group (EPSG): 5937]. (**A**) Northern Canada geology highlighting the regional extent and location of rocks considered to be part of the Franklin LIP. The box on Victoria Island indicates the location of (B). (**B**) Detailed geology of the Minto Inlier on Victoria Island highlighting the location and distribution of Franklin LIP rocks and sample collection locations.

The Franklin magmatic event is one of the largest preserved LIPs in the geological record (*17*). Sills and dykes of the Franklin LIP (*9*) stretch from Alaska across Arctic Canada, from Victoria Island to Baffin Island and into Greenland (Fig. 1) (*9*, *10*). Some sills previously mapped as part of the 1.27-Ga Mackenzie magmatic event (*18*) are now recognized as part of the Franklin LIP, extending the coverage of the LIP farther south to Great Slave Lake (*19*). Assuming that these intrusions fed an extrusive continental flood basalt province that was later eroded away, the outline of this area traces a half circle that covers >5 million km^2^ of North America with dykes radiating away from a focal point north of Banks Island (Fig. 1). If there was a conjugate margin of similar area radiating away in the opposite direction that later rifted away, potentially in Siberia (*20*), then the Franklin LIP would be of comparable size to the ~11 million km^2^ Central Atlantic Magmatic Province (CAMP) and much larger than the 4 million km^2^ Siberian traps (*17*).

**Fig. 2.**
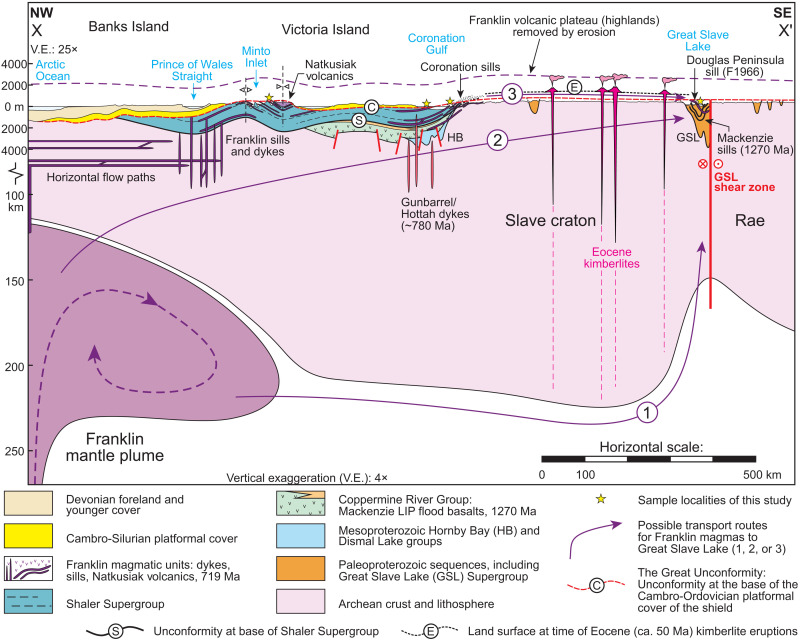
Schematic cross section of the Franklin LIP and starting plume head, and surrounding geological units. The section illustrates the emplacement of the Franklin LIP and three possible paths for sill emplacement in the Great Slave Lake area. Victoria Island sills and dykes may have similarly been emplaced via horizontal flow paths through the lithosphere. Location of cross section *X*-*X′* is indicated in Fig. 1A. Dashed purple line depicts an estimate of the original Franklin LIP volcanic plateau thickness before erosion. Modern elevation on the *y* axis. Reconstructed paleosurfaces are positioned relative to the modern topography and do not indicate absolute paleoelevations at past times. NW, northwest; SE, southeast.

The Franklin LIP was emplaced on the Arctic margin of Laurentia before the rifting of Siberia (*20*) and directly after rifting of North China from the northwest Cordillera (*21*), creating an open margin. During an earlier stage of rifting of the Cordilleran margin, the ca. 778-Ma Gunbarrel LIP was emplaced from Wyoming to Yukon and across the western Slave craton (Fig. 2) (*22*). The Gunbarrel LIP may have covered much of western North America with basalt, but the extrusive component is only preserved in narrow, post–778-Ma rift basins in the Mackenzie Mountains. In the Yukon, these late Tonian rift basins were reactivated by Franklin-age magmatism (*4*).

In Arctic Canada, the Franklin LIP intrudes Archean to Paleoproterozoic basement and Paleoproterozoic to Neoproterozoic sedimentary rocks and is locally preserved as extensive basalt flows on Victoria Island (Fig. 2). Initial observations of dykes feeding multiple sills at different stratigraphic levels and crosscutting extrusive parts of the Franklin LIP led to the conclusion that the emplacement of the sills, dykes, and flows was closely linked in time (*23*). Franklin sills range from 1 to 100 m thick and show geochemical evidence for both fractional crystallization and crustal contamination (*23*–*25*). Much of the Franklin LIP has been eroded away, but to capture the geographic and compositional range of the Franklin LIP, samples for the present study include sills and dykes from the Amundsen Basin (Minto Inlier on Victoria Island, the Brock Inlier on the southern Amundsen Gulf, and the Coppermine area in Coronation Gulf), Great Slave Lake, and the Cumberland Peninsula on Baffin Island (Fig. 1).

The best exposures of the intrusive and extrusive relationships of the Franklin LIP units are on Victoria Island where the bulk of the volcanostratigraphic relationships has been established, also providing a geochemical framework for the rest of the Franklin LIP (*23*, *25*–*27*). On Victoria Island, the >4-km-thick Shaler Supergroup includes organic-rich shale and sulfate evaporites (*28*, *29*) and is succeeded by a >1-km-thick flood basalt sequence, the Natkusiak Formation (*30*). In southwestern exposures, the Shaler Supergroup and Natkusiak Formation contact is conformable, but to the northeast, the contact is unconformable and marked by sedimentary breccia (*29*, *31*). This northeastward pinch-out of the Shaler Supergroup has been interpreted to record doming of the crust associated with the impingement of the Franklin LIP mantle plume (*31*). The Natkusiak Formation consists of basal rubbly flows, volcaniclastic units, and thick sheet flows of basalt (*26*, *32*). Franklin sills intruded the basal Natkusiak Formation and have been linked stratigraphically with crosscutting dykes and geochemically with different extrusive flow types (*11*, *26*, *27*), providing coarse-grained equivalents to the extrusive units that can be more easily and reliably dated. Evidence for glaciation has not been documented below or within the Natkusiak Formation, and the sub-Cambrian unconformity (*33*) precludes further stratigraphic constraints on the relationship between the Franklin LIP and the Sturtian glaciation. Gentle folding of the sub-Cambrian stratigraphy on Victoria Island (Figs. 1 and 2) may be associated with plume emplacement, which can generate intraplate deformation and surface topography (*34*).

In the East Arm of Great Slave Lake, the ~50- to 250-m-thick Douglas Peninsula sill intrudes sedimentary and volcanic rocks of the Paleoproterozoic Great Slave Supergroup and Et-then Group (Fig. 2) (*19*). Three potential paths for how the Franklin LIP mantle plume could have fed the Douglas Peninsula sill on Great Slave Lake are shown on Fig. 2: path 1, a plume-fed mantle flow around the keel of the Slave craton at depth; path 2, southward propagating dykes (parallel to the plane of the section) through the crust of the Slave craton; and path 3, southward continuation of the original sill province in the Proterozoic cover of the Slave craton. In each of these scenarios, we assume that these intrusions were linked to flows that mantled the surface with Franklin-age extrusive volcanic rocks, which were later removed by exhumation and erosion below the sub-Cambrian unconformity.

### Geochemical classification of Franklin LIP rocks

Major and trace element signatures of mantle sources and crustal contamination distinguish emplacement of earliest plume head magmas from later stages of continental flood basalt magmatism [e.g., (*15*, *35*)]. Similarly, the Franklin LIP has been categorized into type 1 and type 2 magmatic phases on the basis of TiO_2_ weight % (wt %) and light rare earth element (LREE) to heavy rare earth element (HREE) ratios (*25*, *36*). This classification and stratigraphy for the Franklin LIP provide a framework for linking our dates with the spatial extent and relative ages of different magma types.

Most of the Franklin LIP has tholeiitic compositions of 45 to 51 wt % SiO_2_ and 6 to 11 wt % MgO (*24*, *27*). Low-Ti [<1.2 wt % TiO_2_; (*36*)] type 1 intrusive and volcanic rocks are stratigraphically below or are crosscut by the younger, high-Ti type 2 igneous rocks and tend to have incorporated more crustal material, resulting in relatively lower Nb/La ratios, enriched LREEs and steeper REE slopes, and lower initial ε_Nd_ (−6.1 to −0.8) and ε_Hf_ (−4.3 to +4.6) values (*27*, *36*). Type 1 rocks also include olivine-rich cumulates and generally have higher wt % MgO (*25*, *27*).

Type 2 rocks are generally more differentiated; are dominated by clinopyroxene and plagioclase; and have lower Ce/Yb ratios, higher Nb/La ratios, and higher initial ε_Nd_ values (*27*). Type 2 Franklin sills and upper Natkusiak sheet flow basalts have values of ε_Nd_ = +1 to +8.8 and ε_Hf_ = +4.7 to +15.8, which indicate lesser degrees of contamination by continental crust (*27*). Type 1 rocks have been associated with early and limited magmatism, while the younger type 2 rocks have been linked to voluminous melting and the main phase of flood volcanism (*26*, *27*), making them the main targets of this study.

## RESULTS

### Trace element and isotope geochemistry

Major element compositions along with minor and trace element concentrations are presented in table S1. Normalized minor and trace element concentrations are plotted in fig. S1. The 93JP-71 samples from Victoria Island and F1966 from Great Slave Lake show slightly LREE-enriched, concave-downward REE patterns with negative Eu anomalies (fig. S1). Granitic sample 93JP-71JB shows an enrichment in total REEs relative to the gabbroic sample 93JP-71M, consistent with differentiation. Samples 93JP-93L from the Duke of York Inlier and 14RAT-513A from the Brock Inlier show similar REE patterns to the other 93JP samples and F1966 but are slightly LREE-enriched and more HREE-depleted. In contrast, samples 17RAT-R35B1 from the Coppermine area, FA700408 from Baffin Island, and S8 from Victoria Island have steeper REE patterns, and all show more LREE enrichment than the other samples.

Values for (Ce/Yb)_CH_ (normalized to chondrite) reflect the REE slopes and are lowest for F1966 (1.30) and the 93JP-71 samples (1.60 to 1.84). (Ce/Yb)_CH_ values increase to 2.41 for 14RAT-513A and 2.97 for 93JP-93L. Values for S8, FA700408, and 17RAT-R35B1 range from 3.41 to 7.64.

Values of (Nb/La)_PM_ (normalized to primitive mantle) for the 93JP-71 samples and F1966 are close to 1.0 (0.96 to 1.01 and 1.01, respectively). Sample 14RAT-513A has a slightly lower (Nb/La)_PM_ value of 0.74, and FA700408 has a (Nb/La)_PM_ value of 0.68. Samples 17RAT-R35B1 and S8 have the lowest (Nb/La)_PM_ values of 0.20 and 0.42, respectively.

The ε_Nd_(*t*) values calculated for an age of 719 Ma cover a wide isotopic range (table S2). Samples 17RAT-R35B1 and S8 have similarly negative ε_Nd_ values of −4.87 and −5.10, while all other samples have positive ε_Nd_ values ranging from 1.72 (FA700408) to 6.59 (F1966). Figure S2 plots (Ce/Yb)_CH_ versus ε_Nd_(719 Ma) for the samples in this study compared to data from (*27*).

### Geochronology

**Fig. 3.**
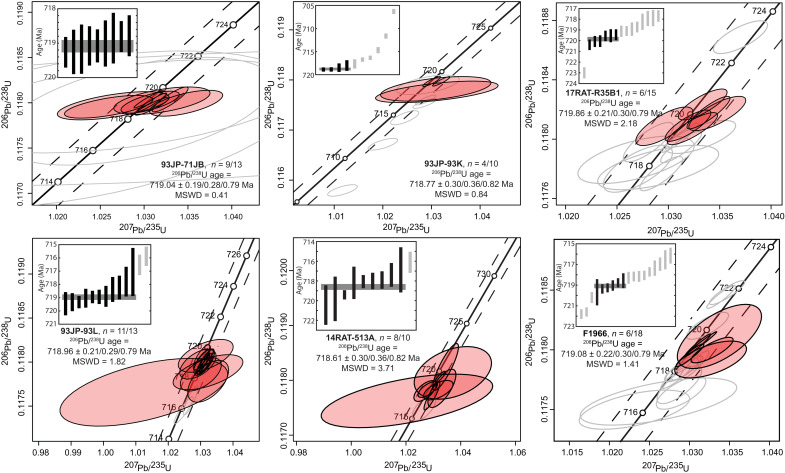
Concordia plots for zircon analyses. Analyses are shown for samples 93JP-71JB, 93JP-93K, 17RAT-R35B1, F1966, 14RAT-513A, and 93JP-93L (clockwise from the top left). The baddeleyite analyses for samples S8 and FA700408 are included in the Supplementary Materials (fig. S3). Concordia plots were modified from IsoplotR (*71*). Gray data ellipses are excluded from the weighted mean dates. All uncertainties are 2σ. Uncertainties for weighted mean dates in this study are reported as ± *X*/*Y*/*Z*, where *X* represents internal error only, *Y* includes tracer calibration uncertainties, and *Z* includes both tracer calibration and decay constant uncertainties for comparisons with different isotopic chronometers. Four low precision analyses are excluded from the plots for 93JP-71JB—the excluded data overlap the plotted analyses within uncertainty (Fig. 4A).

**Fig. 4.**
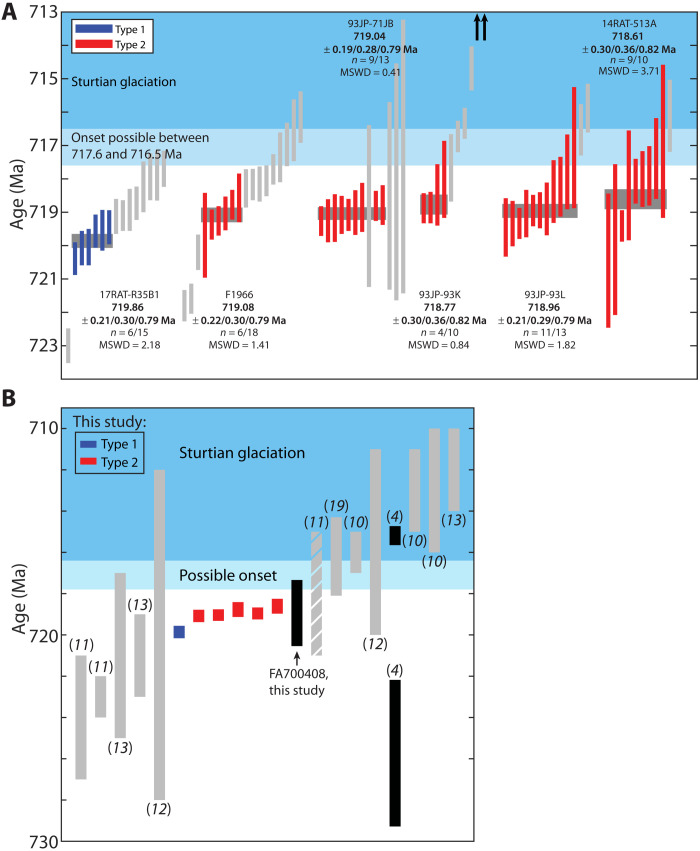
Geochronology of the Franklin LIP. (**A**) Ranked plots of the zircon ^206^Pb/^238^U dates for this study. The light blue bar represents the most precise age constraints on the onset of the Sturtian glaciation between 717.4 ± 0.2/0.4 and 716.9 ± 0.4/0.5 Ma ago [2σ,*X*/*Y* uncertainties; (**14**)] Uncertainties presented in (A) are internal errors only (*X* uncertainties), including for onset constraints since analyses were conducted in the same laboratory. Gray data points are excluded from the weighted mean dates. Up arrows for sample 93JP-93K indicate analyses with Pb loss that are younger than 713 Ma. (**B**) Previous geochronology on the Franklin LIP yielded weighted mean or upper-intercept dates that range from 750 to 710 Ma; analyses from 735 to 710 Ma are plotted (*4*, *10*–*13*, *19*). Previous upper-intercept dates were recalculated with the updated ^238^U/^235^U ratio for terrestrial zircon of 137.818 (*75*) using IsoplotR (*71*). Neoproterozoic ^206^Pb/^238^U dates would not change within uncertainty and were not recalculated from their original references. Details for the dates can be found in the Supplementary Materials (table S4). Light gray coloration indicates either single-grain or bulk baddeleyite analyses; the single diagonally striped sample (*11*) included both bulk zircon and bulk baddeleyite analyses. Black-filled rectangles indicate results for S8 and FA700408, both consisting of single-grain baddeleyite analyses. The two rectangles for S8 show the older upper-intercept and younger ^206^Pb/^238^U dates calculated from single-grain baddeleyite analyses in (*4*), while the rectangle shown for FA700408 is the calculated upper-intercept date (95% CI shown). Other results from this study are red or blue, indicating Franklin LIP magma type, and follow the same sample order as in (A). The 2σ uncertainties for selected analyses and onset constraints include internal and tracer calibration errors (*Y* uncertainties) for interlaboratory comparison. .

High-precision U-Pb zircon geochronology yielded weighted mean ^206^Pb/^238^U dates for the samples from Coppermine area (17RAT-R35B1), Minto Inlier (93JP-71JB), Duke of York Inlier (93JP-93K and 93JP-93L), Brock Inlier (14RAT-513A), and Great Slave Lake (F1966) that range between 719.86 ± 0.21 and 718.61 ± 0.30 Ma (Figs. 3 and 4 and tables S3 and S5). Concordia plots for all zircon analyses are presented in Fig. 3, and plots for baddeleyite analyses are included in the Supplementary Materials (fig. S3). Uncertainties for weighted mean dates in this study are reported as ± *X*/*Y*/*Z*, where *X* represents internal error only, *Y* includes tracer calibration uncertainties, and *Z* includes both tracer calibration and decay constant uncertainties for comparisons with different isotopic chronometers. For comparisons of analyses produced using the same isotopic tracers and techniques, only internal errors will be discussed. Tracer calibration uncertainties are included (*Y* error bounds) for interlaboratory comparisons with analyses that used different isotopic tracer solutions.

Nine fractions of 93JP-71JB yielded a weighted mean ^206^Pb/^238^U date of 719.04 ± 0.19/0.28/0.79 Ma (*n* = 9 of 13, mean square weighted deviation or MSWD = 0.41)—the four excluded dates overlap the other dates within uncertainty but have much larger uncertainties and did not meaningfully contribute to the weighted mean. Sample 93JP-93K yielded an array of concordant and discordant data that plot along a line defined by recent Pb loss (Fig. 3). The weighted mean of the four oldest, most concordant analyses provided a ^206^Pb/^238^U date of 718.77 ± 0.30/0.36/0.82 Ma (*n* = 4 of 10, MSWD = 0.84). Sample 93JP-93L also showed evidence for Pb loss. Excluding two younger analyses resulted in a single, statistically coherent population with a weighted mean ^206^Pb/^238^U date of 718.96 ± 0.21/0.29/0.79 Ma (*n* = 11 of 13, MSWD = 1.82). Sample 14RAT-513A had metamict zircon grains. A weighted mean of the nine oldest analyses produced a date of 718.61 ± 0.30/0.36/0.82 Ma (*n* = 9 of 10, MSWD = 3.71). Given the metamict nature of the analyzed grains, the excluded younger analysis is likely affected by Pb loss.

Analyses for samples 17RAT-R35B1 and F1966 produced an array of dates along concordia, and each had at least one much older analysis. No single weighted mean date could be determined from the data collected. Considering only the analyses that overlapped within uncertainty and produced an MSWD consistent with a single population, the older grains for the main data cluster for sample 17RAT-R35B had a weighted mean of 719.86 ± 0.21/0.30/0.79 Ma (*n* = 6 of 15, MSWD = 2.18) and the six older analyses that overlapped within uncertainty for F1966 produced a weighted mean of 719.08 ± 0.22/0.30/0.79 Ma (*n* = 6 of 18, MSWD = 1.41).

Baddeleyite analyses for sample FA700408 (Baffin Island) generally plotted below concordia, although two grains overlapped concordia within uncertainty (fig. S3). The upper-intercept date for the three analyses constrained to a present-day Pb-loss line was 718.94 ± 1.60 Ma [95% confidence interval (CI), *n* = 3 of 4, MSWD = 1.1]. A single zircon analysis from this sample was excluded for its large uncertainty. Lastly, a date for sample S8 (Victoria Island) was previously published in (*4*) but was recalculated with updated spike calibrations and U blank estimates for Fig. 4B, to be consistent with the data from the present study. The revised U-Pb baddeleyite ^206^Pb/^238^U date for the S8 sample is 715.19 ± 0.41 Ma (*n* = 5 of 7, MSWD = 1.99). The calculated upper-intercept date for S8 is 725.73 ± 3.55 Ma (95% CI, *n* = 7 of 7, MSWD = 1.1).

### Thermochronology

To investigate the postemplacement thermal and exhumation history, (U-Th)/He dates were obtained for sample F1966 in the southern part of the study region. A total of six zircon and six apatite (U-Th)/He (ZHe and AHe) dates were determined (table S6). ZHe dates span from 42 ± 4 to 641 ± 24 Ma (date uncertainty is the propagated 2σ analytical uncertainty) and are negatively correlated with effective uranium concentration (eU), a radiation damage proxy (fig. S5). AHe dates are Phanerozoic. Thermal histories able to explain these data were investigated with inverse thermal history modeling (see Supplementary Text, fig. S5, and table S7).

## DISCUSSION

### Geochemistry of type 1 and type 2 Franklin LIP rocks

The relatively high (Nb/La)_PM_ and low (Ce/Yb)_CH_ ratios for the 93JP-71, 93JP-93, F1966, and 14RAT-513A samples, coupled with positive initial ε_Nd_(*t*) values and their geographic location, suggest that these samples are from the type 2 high-volume phase of Franklin magmas. Samples 17RAT-R35B1 and S8 are distinct from the other samples in their negative initial ε_Nd_(*t*) values. The two samples—along with FA700408—show steeper REE patterns (fig. S1), with high (Ce/Yb)_CH_ (3.41 to 7.64) and lower (Nb/La)_PM_ values (0.20 to 0.42), reflecting more crustal contamination, which is consistent with a type 1 classification. A plot of (Ce/Yb)_CH_ versus ε_Nd_ shows that samples 93JP-93, 93JP-71, 14RAT-513A, and F1966 either plot close to or overlap with values for type 2 rocks, while S8 and 17RAT-R35B1 are more similar to type 1 rocks (fig. S2). Sample FA700408 shows incorporation of more crustal material in terms of LREE enrichment and a higher ε_Nd_(*t*) value. These values could reflect a separate high ε_Nd_ mantle source like Southern type 1 basalts (see Supplementary Text) but with considerably more crustal contamination.

### Interpretation of the U-Pb dates

We interpret the weighted mean ^206^Pb/^238^U dates for samples 93JP-71JB, 93JP-93K, 93JP-93L, and 14RAT-513A to reflect the intrusion and crystallization age of Franklin sills between 719.04 ± 0.19 and 718.61 ± 0.30 Ma ago (Fig. 4A and table S3). These are high-precision age constraints for the younger and more voluminous type 2 set of Franklin LIP magmas. Samples 17RAT-R35B1 and F1966 yielded more ambiguous results, with a range of dates along concordia. In the context of the other dated samples, the oldest analyses seen in 17RAT-R35B1 and F1966 are taken to represent either antecrysts or, possibly, Pb implantation in the grain domains analyzed following annealing and chemical abrasion; the chemical abrasion process preferentially leaches away the more damaged, U-rich domains, and residual lower U domains could include implanted Pb, resulting in reversely discordant analyses in extreme cases (*16*, *37*). These samples both have prismatic crystals that commonly have melt channels parallel to their *c* axes and simple broad zoning in cathodoluminescence (CL) images, such that we consider it unlikely that most grains contain inclusions of older grains at their centers. However, some grains from F1966 have convolute and irregular zoning in CL that could point to metamictization and/or partial resorption of older, antecrystic zircon. Antecrystic zircon grains in mafic systems have been attributed to the fact that in dry and fast-cooling magmas, zircon is relatively difficult to dissolve, and so the resulting dyke or sill may preserve older populations of zircon that do not record its emplacement age (*38*). We therefore interpret the weighted mean ^206^Pb/^238^U date of the oldest population of analyses with an MSWD representing a single population—excluding the older outliers as inherited antecrysts—in samples 17RAT-R35B1 and F1966 as the best estimate of the age of intrusion (719.86 ± 0.21 and 719.08 ± 0.22 Ma, respectively). Following this interpretation, the younger analyses in these samples reflect pervasive Pb loss. While the range of dates observed in these samples preclude a definitive age interpretation, our preferred weighted mean dates are consistent with the dates from the four samples with simpler U-Pb systematics and the stratigraphic order of the intrusive rocks (i.e., the date from type 1 sample 17RAT-R35B1 is older than the dated type 2 intrusive rocks).

There are several petrological and geochemical reasons to ascribe most of the geological variance in the measured U-Pb dates to Pb loss, including high uranium concentrations, age, accumulated radiation damage, and metamictization of the crystal lattice. These phenomena are directly confirmed in the unusually high solubility (>95% dissolution) of the zircon grains during chemical abrasion for most samples. Hence, our interpretation of the crystallization age of the sills as represented by the oldest major cluster of ^206^Pb/^238^U dates is necessarily distinct from strategies pursued in younger silicic tuffs or interbedded volcaniclastic strata within flood basalt lava piles, where Pb loss can be minor and readily mitigated, while crystal recycling and detrital inheritance are often profound [e.g., (*3*)].

We consider the zircon dates from this study to provide the best estimate of the timing and duration of Franklin LIP magmatism because the data come from an internally consistent dataset of high-precision, chemical abrasion analyses spanning a large portion of the Franklin LIP’s geographic range. All of our zircon weighted mean dates—excluding the type 1 17RAT-R35B1 sample—overlap within 2σ uncertainty, indicating broadly synchronous emplacement of the Franklin LIP type 2 intrusions and volcanic rocks. The data capture a snapshot of emplacement for voluminous type 2 rocks spanning 0.47 ± 0.37 Ma (2σ uncertainties added in quadrature). The interpreted older date from 17RAT-R35B1, which has geochemistry more representative of type 1 rocks, is consistent with the crosscutting relationships constraining type 1 to be older than type 2 intrusions and volcanic rocks. Together with the type 2 rocks dated in this study, the duration of emplacement captured is 1.25 ± 0.37 Ma (2σ uncertainties added in quadrature). If this range captures the main emplacement period of the Franklin LIP, then the duration of emplacement would be similar in tempo with other well-dated LIPs [e.g., (*3*, *15*)].

Our baddeleyite U-Pb results are somewhat more complex. The published baddeleyite date from sample S8 is based on single-grain baddeleyite measurements conducted in the same laboratory as this study. The geochemistry of sill S8, which we present here, is more consistent with the criteria for older Northern type 1 rocks, but the recalculated weighted mean ^206^Pb/^238^U date for this sample (715.19 ± 0.41 Ma) is younger than our zircon dates from other samples (719.86 ± 0.21 to 718.61 ± 0.30 Ma). We suspect that this younger date may reflect more extensive Pb loss, which can be difficult to distinguish from concordant analyses if uncertainties overlap with concordia. If the dates for S8 are discordant because of Pb loss, then the upper-intercept date would more accurately represent the age of crystallization; however, the calculated upper-intercept date for S8 is 725.73 ± 3.55 Ma, which overlaps in uncertainty with previous upper-intercept dates (*11*) but is older than the zircon dates obtained in this study. The offset between zircon and baddeleyite dates may reflect additional complexities in interpreting baddeleyite dates due to factors such as intragrain Pb migration (*39*), excess ^207^Pb due to ^231^Pa enrichment or ^222^Rn loss (*40*), and Pb loss (*41*).

Baddeleyite separated from a sample (FA700408) from Baffin Island in this study produced largely discordant analyses, but the weighted mean ^206^Pb/^238^U value of the two analyses closest to concordia would give a date closer to 712 Ma, which is similar to previous baddeleyite geochronology in the same area [e.g., (*10*, *13*)]. If, instead, the dates were considered discordant because of Pb loss and an upper-intercept age was calculated for the analyses, then the date for the sample would be 718.94 ± 1.60 Ma, largely consistent with the dates obtained from zircon and more consistent with established time scales of emplacement for LIPs (*3*).

The range of zircon dates in our samples is much shorter than the ~40-Ma range in dates previously obtained on the Franklin LIP, which was a result of both higher uncertainties in most of the published data and a wider range of measured dates. Most of the existing dates are from single-grain or bulk (multiple grains) baddeleyite analyses, and one analysis [“Lower Sill” sample from Victoria Island (*11*)] involved both bulk zircon and bulk baddeleyite fractions. These analyses predate the development of the chemical abrasion method for addressing Pb loss in zircon (*16*), and there is now no chemical abrasion approach for treating Pb loss in baddeleyite [cf. (*41*)]. Previously published dates that are younger than the dates obtained in this study may therefore be affected by postcrystallization Pb loss. Interlaboratory biases and systematic errors in different tracer calibrations further complicate direct comparisons between our results and published data. We consider the zircon dates, which are based on a single, well-understood mineral system, to provide the most robust estimate of the timing and duration of Franklin LIP magmatism.

### Temporal relationship to onset of the Sturtian Snowball Earth

**Fig. 5.**
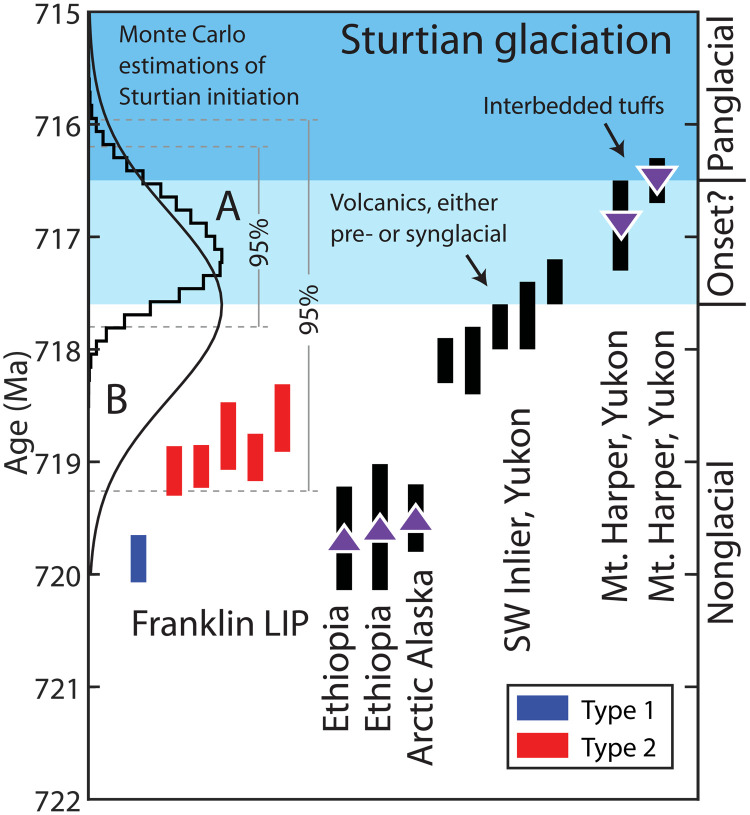
Stratigraphic constraints on the onset of the Sturtian glaciation. Franklin LIP data from this study compared to existing constraints on the onset of the Sturtian glaciation (*14*, *47*, *48*, *76*). Because all analyses used the same technique and same isotopic tracer, only internal uncertainties were plotted for comparison. Purple arrowheads pointing up indicate dates that provide maximum constraints on the age of onset (i.e., glaciation onset must have occurred at a younger date) and arrowheads pointing down indicate minimum age constraints on onset. The ages of volcanic tuffs interbedded with glacial diamictite provide a definitive age by which glaciation must have been initiated. The maximum age bound on onset is still uncertain; the shown analyses bracketing onset are from volcanic sequences that could be subaerial or subglacial, with the possibility of onset being up to several 100 ka before. Previous studies have used Monte Carlo models to estimate onset ages of (**A**) 717.1 + 0.7/− 0.9 Ma (*47*) and (**B**) 717.61 ± 1.65 Ma (*48*). SW, southwest.

The emplacement age of the Franklin LIP, between 719.86 ± 0.21 and 718.61 ± 0.30 Ma ago, is older than previous constraints for the onset of the Sturtian glaciation between 717.4 ± 0.2 and 716.9 ± 0.4 Ma ago [also analyzed in the Boise State University (BSU) Isotope Geology Laboratory using the same tracer calibration (**14**)] by 0.9 to 1.6 Ma (Figs. 4 and 5). Onset is constrained by dates on ca. 718.1- to 717.4-Ma rhyolite flows in member D of the Mt. Harper Volcanic Complex. These rhyolite flows underlie a basalt flow in member E with wrinkled and ropey textures (*42*) and are unconformably overlain by glacial diamictite of the Eagle Creek Formation, which has interbedded ca. 716.9- to 716.5-Ma volcanic tuffs (*14*). The ropey texture, hyaloclastic breccia, bulbous isolated pillow basalts, and lack of obvious subaqueous textures in member E were interpreted to record subaerial eruption (*42*), consistent with predating global glacial onset (*14*); however, similar tubular and bulbous structures, isolated pillows, and ropey textures have been described in subglacial settings (*43*, *44*), such that the subaerial and preglacial interpretation is nonunique, and member E alternatively may have been emplaced in a subglacial setting in an environment like Antarctica’s dry valleys. Given these limitations, the interpreted constraint on the onset of the Sturtian glaciation based on the ca. 717.4-Ma volcanic rocks should only be taken as the minimum bound on the maximum age of Sturtian onset, meaning that onset could be as old or older than this constraint depending on whether the volcanic rocks are preglacial or synglacial. Additionally, it could have taken up to several 100 ka after the ocean froze over for continental ice sheets to thicken sufficiently to flow and produce glacial structures at their periphery (*45*, *46*), further delaying observable evidence of glaciation in the rock record from onset.

Other strict age constraints on the maximum age for the initiation of the Sturtian glaciation come from Ethiopia, South China, and Arctic Alaska and have all yielded ages of ca. 719 to 720 Ma in preglacial strata (Fig. 5) (*47*–*49*). Glacial onset ages have been extrapolated in Monte Carlo simulations estimating sedimentation rates to the base of the glacial diamictite using the maximum age constraints from Ethiopia and South China and have yielded ages of 717.1 + 0.7/− 0.9 Ma [95% CI (*47*)] and 717.61 ± 1.65 Ma [95% CI (*48*)]. These estimates are consistent with onset between 717.4 ± 0.2 and 716.9 ± 0.4 Ma ago based on constraints from the Mt. Harper Group. In comparing the maximum constraint on onset (717.4 ± 0.2 Ma) and the minimum age bound on the younger, high-volume type 2 magmas (14RAT-513A, 718.61 ± 0.30 Ma), Franklin LIP emplacement preceded the onset of glaciation by 1.21 ± 0.36 Ma (2σ uncertainties added in quadrature).

### Initiation of Snowball Earth

Changes in weatherability and volcanic outgassing related to plate tectonics and paleogeography provide a first-order control on Earth’s long-term climate through the silicate weathering feedback (*50*). Snowball Earth represents a failure of this thermostat and is initiated once a critical threshold is reached for runaway ice-albedo feedback (*50*). The weathering of continental flood basalts in particular has been hypothesized as an important sink for CO_2_ because of their large surface areas and highly reactive mafic lithologies (*51*–*53*). Furthermore, modern river data demonstrate that CO_2_ consumption is highest in basaltic watersheds in the warm, wet tropics (*52*, *54*). Models and proxy data for *p*CO_2_ following LIP emplacement show that warming from the radiative effects of the initial input of CO_2_ into the atmosphere can be counterbalanced by silicate weathering, with peaks of LIP-associated weatherability within ~1 to 2 Ma of emplacement (*2*, *53*, *55*), after which time the development of regolith and soil shielding will decrease weatherability (*54*, *56*).

Despite the sink for CO_2_ that LIPs provide on million-year time scales, many LIPs are not associated with glaciation. The Siberian Traps were comparable in size to the Franklin LIP, and the preceding Mackenzie LIP may have been even larger, but neither resulted in a Snowball Earth episode. The lack of correlation between multimillion-year glacial events such as the Permo-Carboniferous glaciations and LIPs can be expected as a result of several factors, including the background climate state [e.g., (*5*)], the paleolatitude of the LIP (*55*), the composition of the country rocks that the LIP is emplaced in (*2*), and even the paleogeography of the continents at the time of emplacement [e.g., (*56*)]. The Siberian Traps and CAMP were emplaced during warm background climate conditions on an ice-free Earth (*5*, *57*), increasing the barrier to runaway glaciation. Weathering also proceeded slowly for the Siberian Traps because of its emplacement at high latitudes (*55*). The Mackenzie LIP was emplaced in an intracontinental setting in a subsiding basin (*18*, *58*), decreasing its topographic relief and weathering impact. In contrast to other LIPs, the emplacement of the Franklin LIP occurred in conjunction with several factors that increased global weatherability and allowed for runaway cooling on a 1- to 2-Ma time scale (*2*, *53*, *55*).

Paleogeography was likely an important factor contributing to increased weatherability and a cool Cryogenian climate due to an abundance of continents after supercontinent breakup (*8*, *56*, *59*), LIPs with mafic lithologies (*6*, *53*), and arc terranes with high relief [e.g., (*60*–**62**)], all at low latitudes in the equatorial rain belt (*63*). In addition, it has been suggested that reduced continental volcanic arc activity during supercontinent breakup could have led to lower CO_2_ outgassing (*64*), further decreasing global temperatures and the threshold for glaciation.

Critical to weatherability is the generation of topography [e.g., (*55*, *61*, **62**)]; if a continental flood basalt province is emplaced at low relief, then it will likely be buried and not greatly increase global weatherability, as happened with the burial of the CAMP during the rifting of the Atlantic. If, instead, a LIP is associated with uplift and exhumation and creates volcanic highlands, then there is strong potential for a rapid increase in global weatherability (*53*, *55*). Studies of plume-head interactions with the lithosphere argue that, as the plume head flattens because of impact with the lithosphere, early uplift at the plume axis is followed by subsidence while the margins of the plume head, possibly out to distances of ~2000 km, should experience progressive uplift (*65*).

On Victoria Island, >500 km from the plume center, an unconformity below subareal basalt flows of the Natkusiak Formation has been interpreted to record the doming of the crust associated with the impingement of the Franklin LIP mantle plume (*31*), supporting the hypothesis that the Franklin LIP generated dynamic topography and created volcanic highlands rather than submerged lowlands associated with sedimentation and burial. The Natkusiak Formation exhibits prehnite-pumpellyite facies metamorphism attributed to an elevated geotherm and the presence of an overlying volcanic succession that was originally >2 km (*32*). The generation of topography due to emplacement of the Franklin LIP followed by its erosion is consistent with coupled Sr and Nd isotopes from carbonates and mudstones from the adjacent margins that document a Cryogenian flux of juvenile material (*7*).

At the southern edge of the study area, Douglas Peninsula sill sample F1966 gives Cryogenian (U-Th)/He zircon dates that alone do not require substantial exhumation immediately after Franklin LIP emplacement; both immediate exhumation from shallow emplacement and shallow burial to temperatures <150°C following emplacement are permissible (fig. S5). However, the paucity of sedimentary Cryogenian to Ediacaran units across the wider study area leads us to conclude that the burial scenario is unlikely and that broad volcanic highlands, much like modern Ethiopia, covered a massive portion of tropical Laurentia (Fig. 2 and fig. S5).

Dynamic topography resulting from plume-head impact is expected to persist on the order of 10 Ma (*65*), and basaltic highlands associated with the Gunbarrel and Franklin LIPs would have contributed to high global weatherability at the Tonian-Cryogenian boundary [e.g., (*60*, *62*)]. Coupled climate-weathering models predict that the removal of recent flood basalt provinces without any prescribed topographic change would change steady-state *p*CO_2_ by >100 parts per million (*62*), demonstrating the potential for cooling associated with the weatherability of LIPs. Thus, the hypothesis that increased global weatherability associated with the tropical emplacement of the Franklin LIP contributed to the onset of the Sturtian Snowball Earth predicts runaway ice-albedo feedback within ~1 to 2 Ma; this is consistent with the geochronology presented here.

There have been multiple factors proposed for shorter-term changes of planetary albedo or radiative forcing that could have also pushed Earth’s climate toward the threshold for a Snowball glaciation, including volcanic aerosols (*5*) or biogeochemical changes [e.g., (*59*, *66*)]. Albedo perturbations should initiate glaciation almost immediately because of the strong forcings but short residence times of aerosols (*5*), and biogeochemical changes would likely cause cooling on time scales of 10^3^ to 10^4^ years (*66*). The timeline presented here is inconsistent with direct, short-term (<100 ka) connections between peak magmatism of the Franklin LIP and the onset of the Sturtian glaciation.

The data presented provide the first high-precision ages on the main, high-volume phase of the Franklin LIP (type 2 eruptions) between 719.08 ± 0.22 and 718.61 ± 0.30 Ma ago. On the basis of the approximately 10 to 90% split between type 1 and type 2 rocks characterized in the Natkusiak Formation (*27*), the geochronology of the type 2 phase is estimated to capture ≥90% of Franklin LIP magmatism. The best estimates on the onset of the Sturtian Snowball Earth glaciation suggest that it began ~0.9 to 1.6 Ma after peak Franklin LIP volcanism. Geology and thermochronology at the northern and southern edges of the study area, respectively, are compatible with postemplacement exhumation corresponding to development of Tonian-Cryogenian volcanic highlands in Cordilleran and Arctic Laurentia. The apparent lag time and generation of topography are consistent with an increase in global weatherability as a primary trigger for onset of glaciation. These results tighten the relationship between the largest Neoproterozoic episode of magmatism and one of the most extreme episodes of climate change in the geologic record.

## MATERIALS AND METHODS

Whole-rock major element, trace element, and Sm-Nd isotope geochemistry were performed for all samples in addition to gabbro sample S8, the source of the most recent high-precision U-Pb baddeleyite date on the Franklin LIP (*4*). Major and trace element concentrations for samples FA700408 and F1966 were measured at the California Institute of Technology following the procedure in (*67*). A Retsch planetary ball mill (PM 100) with agate grinding containers was used for powdering whole-rock samples, and a Claise Eagon 2 fluxer was used for fusing the powders into glass beads using lithium tetraborate as a flux. Major elements were measured on the glass beads using a 4-kW Zetium Panalytical x-ray fluorescence analyzer. Trace elements were measured using solution inductively coupled plasma mass spectrometry (ICPMS) following the methods in (*68*).

The other six samples (93JP-71JB, 93JP-71M, 93JP-93L, S8, 14RAT-513A, and 17RAT-R35B1) were powdered and analyzed at Hamilton College following the procedure in (*69*). Rocks were cut with a diamond saw and powdered in a SPEX 8530 ShatterBox using an alumina ceramic dish at University of California, Santa Barbara (UCSB) and then repowdered at Hamilton College using a Rocklabs alumina ring mill. The powders were doubly fused to homogenize the sample and then analyzed using a Thermo ARL PERFORM’X x-ray fluorescence spectrometer. Trace elements were measured using laser ablation–ICPMS on the fused glass beads following the procedures in (*70*).

Mineral separation for the U-Pb analyses was done at the Harvard University and UCSB. Because most samples were only hand samples from archive, mineral separation procedures were tailored to maximize potential yield of zircon and baddeleyite grains. All samples were hand-sledged into chips that were ≤1 cm^3^ and then pulsed in 1- to 2-s intervals in a SPEX 8530 ShatterBox followed by sieving for the <500-μm fraction. The <500-μm fraction for each sample was handwashed in 5-liter beakers to remove fine material and dried under heat lamps or in low-temperature ovens before being run on the Frantz magnetic separator. Highly magnetic minerals were initially screened for and removed using a hand magnet, and then, samples were typically run twice, first at 0.3 A and 20° tilt and the second time at 0.6 A and 20° tilt. If less than 50 g of sample were left after the first run on the Frantz, then the sample was not run a second time. If the sample was mostly magnetic, then the tilt angle was increased by 5° to 10° to ensure that no nonmagnetic grains were carried by the flow of magnetic grains. Heavy-liquid density separation using methylene iodide was the last step for isolating the dense mineral fraction of zircon and baddeleyite. Zircon and baddeleyite grains were hand-picked for each sample from this final fraction.

Geochronological analyses were done at the BSU. Zircon crystals were mounted in epoxy, polished to expose grain cores, and imaged using a CL detector. U-Pb dates were obtained using the CA-ID-TIMS procedure developed in (*16*). Dissolution, spiking, and column chemistry procedures are described in more detail in the Supplementary Materials. Baddeleyite geochronology followed the same procedure except that grains were not subjected to chemical abrasion and were instead fluxed in 3.5 M HNO_3_ for 20 to 30 min on a hot plate and then sonicated for the same amount of time before being rinsed and loaded into microcapsules for dissolution with spike. Concordia and upper-intercept dates were plotted using IsoplotR (*71*).

Sm and Nd isotope geochemistry was done at the Isotope Geology Laboratory at the BSU. Powdered samples were spiked and dissolved at 220°C for 18 hours. Detailed column chemistry procedures are described in the Supplementary Materials. Samples were analyzed on the IsotopX Phoenix X62 TIMS. (U-Th)/He analysis was done at the Thermochronology Research and Instrumentation Lab at the University of Colorado Boulder following methods described in the Supplementary Materials.

## Supplementary Materials

### This PDF file includes:

Supplementary Text Figs. S1 to S5 Tables S1 to S7 References

## References

[1] P. F. Hoffman, D. S. Abbot, Y. Ashkenazy, D. I. Benn, J. J. Brocks, P. A. Cohen, G. M. Cox, J. R. Creveling, Y. Donnadieu, D. H. Erwin, I. J. Fairchild, D. Ferreira, J. C. Goodman, G. P. Halverson, M. F. Jansen, G. Le Hir, G. D. Love, F. A. Macdonald, A. C. Maloof, C. A. Partin, G. Ramstein, B. E. J. Rose, C. V. Rose, P. M. Sadler, E. Tziperman, A. Voigt, S. G. Warren,Snowball Earth climate dynamics and Cryogenian geology-geobiology. Sci. Adv. 3,e1600983 (2017). 29134193 10.1126/sciadv.1600983 PMC5677351

[2] M. T. Jones, D. A. Jerram, H. H. Svensen, C. Grove,The effects of large igneous provinces on the global carbon and sulphur cycles. Palaeogeogr. Palaeoclimatol. Palaeoecol. 441,4–21 (2015).

[3] J. Kasbohm, B. Schoene, S. Burgess, Radiometric constraints on the timing, tempo, and effects of large igneous province emplacement, in *Large Igneous Provinces: A Driver of Global Environmental and Biotic Changes*, R. E. Ernst, A. J. Dickson, A. Bekker, Eds. (Geophysical Monograph 255, AGU and Wiley, ed. 1, 2021), pp. 27–82.

[4] F. A. Macdonald, M. D. Schmitz, J. L. Crowley, C. F. Roots, D. S. Jones, A. C. Maloof, J. V. Strauss, P. A. Cohen, D. T. Johnston, D. P. Schrag,Calibrating the cryogenian. Science 327,1241–1243 (2010). 20203045 10.1126/science.1183325

[5] F. A. Macdonald, R. Wordsworth,Initiation of Snowball Earth with volcanic sulfur aerosol emissions. Geophys. Res. Lett. 44,1938–1946 (2017).

[6] Y. Goddéris, Y. Donnadieu, A. Nédélec, B. Dupré, C. Dessert, A. Grard, G. Ramstein, L. M. François,The Sturtian “snowball” glaciation: Fire and ice. Earth Planet. Sci. Lett. 211,1–12 (2003).

[7] G. M. Cox, G. P. Halverson, R. K. Stevenson, M. Vokaty, A. Poirier, M. Kunzmann, Z. X. Li, S. W. Denyszyn, J. V. Strauss, F. A. Macdonald,Continental flood basalt weathering as a trigger for Neoproterozoic Snowball Earth. Earth Planet. Sci. Lett. 446,89–99 (2016).

[8] Y. Donnadieu, Y. Goddéris, G. Ramstein, A. Nédélec, J. Meert,A “snowball Earth” climate triggered by continental break-up through changes in runoff. Nature 428,303–306 (2004). 15029192 10.1038/nature02408

[9] W. F. Fahrig, E. Irving, G. D. Jackson,Paleomagnetism of the Franklin diabases. Can. J. Earth Sci. 8,455–467 (1971).

[10] S. W. Denyszyn, H. C. Halls, D. W. Davis, D. A. D. Evans,Paleomagnetism and U-Pb geochronology of Franklin dykes in high arctic Canada and Greenland: A revised age and paleomagnetic pole constraining block rotations in the Nares Strait region. Can. J. Earth Sci. 46,689–705 (2009).

[11] L. M. Heaman, A. N. LeCheminant, R. H. Rainbird,Nature and timing of Franklin igneous events, Canada: Implications for a Late Proterozoic mantle plume and the break-up of Laurentia. Earth Planet. Sci. Lett. 109,117–131 (1992).

[12] S. J. Pehrsson, K. L. Buchan,Borden dykes of Baffin Island, Northwest Territories: A Franklin U-Pb baddeleyite age and a paleomagnetic reinterpretation. Can. J. Earth Sci. 36,65–73 (1999).

[13] S. W. Denyszyn, D. W. Davis, H. C. Halls,Paleomagnetism and U-Pb geochronology of the Clarence head dykes, Arctic Canada: Orthogonal emplacement of mafic dykes in a large igneous province. Can. J. Earth Sci. 46,155–167 (2009).

[14] F. A. Macdonald, M. D. Schmitz, J. V. Strauss, G. P. Halverson, T. M. Gibson, A. Eyster, G. Cox, P. Mamrol, J. L. Crowley,Cryogenian of Yukon. Precambrian Res. 319,114–143 (2017).

[15] S. E. Bryan, R. E. Ernst,Revised definition of large igneous provinces (LIPs). Earth Sci. Rev. 86,175–202 (2008).

[16] J. M. Mattinson,Zircon U-Pb chemical abrasion (“CA-TIMS”) method: Combined annealing and multi-step partial dissolution analysis for improved precision and accuracy of zircon ages. Chem. Geol. 220,47–66 (2005).

[17] R. E. Ernst, D. P. G. Bond, S. Zhang, K. L. Buchan, S. E. Grasby, N. Youbi, H. El Bilali, A. Bekker, L. S. Doucet, Large igneous province record through time and implications for secular environmental changes and geological time-scale boundaries, in *Large Igneous Provinces: A Driver of Global Environmental and Biotic Changes*, R. E. Ernst, A. J. Dickson, A. Bekker, Eds. (Geophysical Monograph 255, AGU and Wiley, ed. 1, 2021), pp. 1–26.

[18] A. N. LeCheminant, L. M. Heaman,Mackenzie igneous events, Canada: Middle Proterozoic hotspot magmatism associated with ocean opening. Earth Planet. Sci. Lett. 96,38–48 (1989).

[19] W. Bleeker, M. A. Hamilton, U. Söderlund, “A Franklin age (716 Ma) for a large gabbro sill on Great Slave Lake, Northwest Territories, Canada” (Report No. A149, 2013);www.supercontinent.org.

[20] R. E. Ernst, M. A. Hamilton, U. Söderlund, J. A. Hanes, D. P. Gladkochub, A. V. Okrugin, T. Kolotilina, A. S. Mekhonoshin, W. Bleeker, A. N. LeCheminant, K. L. Buchan, K. R. Chamberlain, A. N. Didenko,Long-lived connection between southern Siberia and northern Laurentia in the Proterozoic. Nat. Geosci. 9,464–469 (2016).

[21] J. Ding, S. Zhang, D. A. D. Evans, T. Yang, H. Li, H. Wu, J. Chen,North China craton: The conjugate margin for northwestern Laurentia in Rodinia. Geology 49,773–778 (2021).

[22] S. S. Harlan, L. Heaman, A. N. LeCheminant, W. R. Premo,Gunbarrel mafic magmatic event: A key 780 Ma time marker for Rodinia plate reconstructions. Geology 31,1053–1056 (2003).

[23] W. Baragar, The Natkusiak Basalts, Victoria Island, District of Franklin (Paper 76-1A, Geological Survey of Canada, 1976), pp. 347–352.

[24] J. Dostal, W. R. A. Baragar, C. Dupuy,Petrogenesis of the Natkusiak continental basalts, Victoria Island, Northwest Territories Canada. Can. J. Earth Sci. 23,622–632 (1986).

[25] J. H. Bédard, B. Hayes, M. Hryciuk, C. Beard, N. Williamson, T. A. Dell’Oro, R. H. Rainbird, J. Prince, W. R. A. Baragar, P. Nabelek, D. Weis, B. Wing, J. S. Scoates, H. R. Naslund, B. Cousens, M.-C. Williamson, L. J. Hulbert, R. Montjoie, É. Girard, R. Ernst, C. J. Lissenberg, Geochemical database of Franklin sills, Natkusiak Basalts and Shaler Supergroup rocks, Victoria Island, Northwest Territories, and correlatives from Nunavut and the mainland (Open File Rep. 8009, Geological Survey of Canada, 2016; 1 .zip file, 10.4095/297842).

[26] N. M. B. Williamson, L. Ootes, R. H. Rainbird, J. H. Bédard, B. Cousens,Initiation and early evolution of the Franklin magmatic event preserved in the 720 Ma Natkusiak Formation, Victoria Island Canadian Arctic. Bull. Volcanol. 78,1–19 (2016).

[27] C. D. Beard, J. S. Scoates, D. Weis, J. H. Bédard, T. A. Dell’Oro,Geochemistry and origin of the Neoproterozoic Natkusiak flood basalts and related Franklin sills, Victoria Island Arctic Canada. J. Petrol. 58,2191–2220 (2018).

[28] G. M. Young, The Amundsen Embayment, Northwest Territories; Relevance to the upper Proterozoic evolution of North America, in *Proterozoic Basins of Canada*, F. H. A. Campbell, Ed. (Paper 81-10, Geological Survey of Canada, 1981), pp. 203–218.

[29] R. H. Rainbird, C. W. Jefferson, G. M. Young,The early Neoproterozoic sedimentary Succession B of northwestern Laurentia: Correlations and paleogeographic significance. GSA Bull. 108,454–470 (1996).

[30] R. Thorsteinsson, E. T. Tozer, Banks, Victoria, and Stefansson Islands, Arctic Archipelago (Memoir 330, Geological Survey of Canada, 1962), pp. 85.

[31] R. H. Rainbird,The sedimentary record of mantle plume uplift preceding eruption of the Neoproterozoic Natkusiak flood basalt. J. Geol. 101,305–318 (1993).

[32] C. W. Jefferson, W. E. Nelson, R. V. Kirkham, J. H. Reedman, R. F. J. Scoates, Geology and copper occurrences of the Natkusiak basalts, Victoria Island, District of Franklin, in *Current Research, Part* A (Paper 85–1A, Geological Survey of Canada, 1985), pp. 203–214.

[33] A. M. Durbano, B. R. Pratt, T. Hadlari, K. Dewing,Sedimentology of an early Cambrian tide-dominated embayment: Quyuk formation, Victoria Island Arctic Canada. Sediment. Geol. 320,1–18 (2015).

[34] E. Burov, S. Cloetingh,Controls of mantle plumes and lithospheric folding on modes of intraplate continental tectonics: Differences and similarities. Geophys. J. Int. 178,1691–1722 (2009).

[35] R. W. Carlson,Physical and chemical evidence on the cause and source characteristics of flood basalt volcanism. Aust. J. Earth Sci. 38,525–544 (1991).

[36] T. A. Dell’Oro, “Sr-Nd-Hf-Pb isotope and trace element geochemistry of the Natkusiak Formation continental flood basalts of the Neoproterozoic Franklin large igneous province, Victoria Island, Canada,” thesis, University of British Columbia, Vancouver, Canada (2012).

[37] J. M. Mattinson, C. M. Graubard, D. L. Parkinson, W. C. McClelland, U-Pb reverse discordance in zircons: The role of fine-scale oscillatory zoning and sub-micron transport of Pb, in *Earth Processes: Reading the Isotopic Code*, A. Basu, S. Hart, Eds. (Geophysical Monograph Series, AGU, 1996), vol. 95, pp. 355–370.

[38] L. P. Black, P. D. Kinny, J. W. Sheraton,The difficulties of dating mafic dykes: An Antarctic example. Contrib. Mineral. Petrol. 109,183–194 (1991).

[39] M. Ibanez-Mejia, G. E. Gehrels, J. Ruiz, J. D. Vervoort, M. E. Eddy, C. Li,Small-volume baddeleyite (ZrO_2_) U-Pb geochronology and Lu-Hf isotope geochemistry by LA-ICP-MS. Techniques and applications. Chem. Geol. 384,149–167 (2014).

[40] L. M. Heaman, A. N. LeCheminant,Anomalous U-Pb systematics in mantle-derived baddeleyite xenocrysts from Île Bizard: Evidence for high temperature radon diffusion? Chem. Geol. 172,77–93 (2000).

[41] M. Rioux, S. Bowring, F. Dudás, R. Hanson,Characterizing the U-Pb systematics of baddeleyite through chemical abrasion: Application of multi-step digestion methods to baddeleyite geochronology. Contrib. Mineral. Petrol. 160,777–801 (2010).

[42] P. S. Mustard, C. F. Roots,Rift-related volcanism, sedimentation, and tectonic setting of the Mount Harper Group, Ogilvie Mountains Yukon Territory. Geol. Surv. Can. Bull. 492,92 (1997).

[43] J. A. Stevenson, J. L. Smellie, D. W. McGarvie, J. S. Gilbert, B. I. Cameron,Subglacial intermediate volcanism at Kerlingarfjöll, Iceland: Magma-water interactions beneath thick ice. J. Volcanol. Geotherm. Res. 185,337–351 (2009).

[44] J. L. Smellie,Terrestrial subice volcanism: Landform morphology, sequence characteristics, environmental influences, and implications for candidate Mars examples. GSA Spec. Pap. 453,55–76 (2009).

[45] Y. Donnadieu, F. Fluteau, G. Ramstein, C. Ritz, J. Besse,Is there a conflict between the Neoproterozoic glacial deposits and the snowball Earth interpretation: An improved understanding with numerical modeling. Earth Planet. Sci. Lett. 208,101–112 (2003).

[46] D. Pollard, J. F. Kasting, Climate-ice sheet simulations of neoproterozoic glaciation before and after collapse to snowball Earth, in *The Extreme Proterozoic: Geology, Geochemistry, and Climate*, G. S. Jenkins, M. A. S. McMenamin, C. P. McKay, L. Sohl, Eds. (Geophysical Monograph Series, AGU, 2004), vol. 146, pp. 91–105.

[47] S. MacLennan, Y. Park, N. Swanson-Hysell, A. Maloof, B. Schoene, M. Gebreslassie, E. Antilla, T. Tesema, M. Alene, B. Haileab,The arc of the Snowball: U-Pb dates constrain the Islay anomaly and the initiation of the Sturtian glaciation. Geology 46,539–542 (2018).

[48] Z. Lan, M. H. Huyskens, K. Lu, X. H. Li, G. Zhang, D. Lu, Q. Z. Yin,Toward refining the onset age of Sturtian glaciation in South China. Precambrian Res. 338,105555 (2020).

[49] G. M. Cox, J. V. Strauss, G. P. Halverson, M. D. Schmitz, W. C. McClelland, R. S. Stevenson, F. A. Macdonald,Kikiktat volcanics of Arctic Alaska-Melting of harzburgitic mantle associated with the Franklin large igneous province. Lithosphere 7,275–295 (2015).

[50] J. C. G. Walker, P. B. Hays, J. F. Kasting,A negative feedback mechanism for the long-term stabilization of Earth’s surface temperature. J. Geophys. Res. 86,9776–9782 (1981).

[51] G. Li, J. Hartmann, L. A. Derry, A. J. West, C. F. You, X. Long, T. Zhan, L. Li, G. Li, W. Qiu, T. Li, L. Liu, Y. Chen, J. Ji, L. Zhao, J. Chen,Temperature dependence of basalt weathering. Earth Planet. Sci. Lett. 443,59–69 (2016).

[52] C. Dessert, B. Dupré, J. Gaillardet, L. M. François, C. J. Allègre,Basalt weathering laws and the impact of basalt weathering on the global carbon cycle. Chem. Geol. 202,257–273 (2003).

[53] C. Dessert, B. Dupré, L. M. François, J. Schott, J. Gaillardet, G. Chakrapani, S. Bajpai,Erosion of Deccan Traps determined by river geochemistry: Impact on the global climate and the ^87^Sr/^86^Sr ratio of seawater. Earth Planet. Sci. Lett. 188,459–474 (2001).

[54] J. Hartmann, N. Moosdorf, R. Lauerwald, M. Hinderer, A. J. West,Global chemical weathering and associated P-release—The role of lithology, temperature and soil properties. Chem. Geol. 363,145–163 (2014).

[55] Y. Park, N. L. Swanson-Hysell, L. E. Lisiecki, F. A. Macdonald, Evaluating the relationship between the area and latitude of large igneous provinces and Earth’s long-term climate state, in *Large Igneous Provinces: A Driver of Global Environmental and Biotic Changes*, R. E. Ernst, A. J. Dickson, A. Bekker, Eds. (Geophysical Monograph 255, AGU and Wiley, ed. 1, 2021), pp. 153–168.

[56] Y. Goddéris, G. Le Hir, M. Macouin, Y. Donnadieu, L. Hubert-Théou, G. Dera, M. Aretz, F. Fluteau, Z. X. Li, G. P. Halverson,Paleogeographic forcing of the strontium isotopic cycle in the Neoproterozoic. Gondw. Res. 42,151–162 (2017).

[57] E. L. Taylor, T. N. Taylor, N. R. Cúneo,The present is not the key to the past: A polar forest from the permian of antarctica. Science 257,1675–1677 (1992). 17841167 10.1126/science.257.5077.1675

[58] R. H. Rainbird, R. E. Ernst,The sedimentary record of mantle-plume uplift. Geol. Soc. Am. Spec. Paper 352,227–245 (2001).

[59] D. P. Schrag, R. A. Berner, P. F. Hoffman, G. P. Halverson,On the initiation of a snowball Earth. Geochem. Geophys. Geosyst. 3,1–21 (2002).

[60] Y. Park, N. L. Swanson-Hysell, S. A. MacLennan, A. C. Maloof, M. Gebreslassie, M. M. Tremblay, B. Schoene, M. Alene, E. S. C. Anttila, T. Tesema, B. Haileab,The lead-up to the Sturtian Snowball Earth: Neoproterozoic chemostratigraphy time-calibrated by the Tambien Group of Ethiopia. GSA Bull. 132,1119–1149 (2019).

[61] F. A. Macdonald, N. L. Swanson-Hysell, Y. Park, L. Lisiecki, O. Jagoutz,Arc-continent collisions in the tropics set Earth’s climate state. Science 364,181–184 (2019). 30872536 10.1126/science.aav5300

[62] Y. Park, P. Maffre, Y. Goddéris, F. A. MacDonald, E. S. C. Anttila, N. L. Swanson-Hysell,Emergence of the Southeast Asian islands as a driver for neogene cooling. Proc. Natl. Acad. Sci. U.S.A. 117,25319–25326 (2020). 32973090 10.1073/pnas.2011033117 PMC7568243

[63] Z. X. Li, D. A. D. Evans, G. P. Halverson,Neoproterozoic glaciations in a revised global palaeogeography from the breakup of Rodinia to the assembly of Gondwanaland. Sediment. Geol. 294,219–232 (2013).

[64] N. R. McKenzie, B. K. Horton, S. E. Loomis, D. F. Stockli, N. J. Planavsky, C. T. A. Lee,Continental arc volcanism as the principal driver of icehouse-greenhouse variability. Science 352,444–447 (2016). 27102480 10.1126/science.aad5787

[65] A. M. Friedrich, H. P. Bunge, S. M. Rieger, L. Colli, S. Ghelichkhan, R. Nerlich,Stratigraphic framework for the plume mode of mantle convection and the analysis of interregional unconformities on geological maps. Gondw. Res. 53,159–188 (2018).

[66] E. Tziperman, I. Halevy, D. T. Johnston, A. H. Knoll, D. P. Schrag,Biologically induced initiation of neoproterozoic snowball-Earth events. Proc. Natl. Acad. Sci. U.S.A. 108,15091–15096 (2011). 21825156 10.1073/pnas.1016361108 PMC3174660

[67] C. E. Bucholz, C. J. Spencer,Strongly peraluminous granites across the archean-proterozoic transition. J. Petrol. 60,1299–1348 (2019).

[68] M. J. Lewis, C. E. Bucholz, O. E. Jagoutz,Evidence for polybaric fractional crystallization in a continental arc: Hidden Lakes mafic complex, Sierra Nevada batholith California. Contrib. Mineral. Petrol. 176,1–27 (2021).

[69] D. M. Johnson, P. R. Hooper, R. M. Conrey,XRF analysis of rocks and minerals for major and trace elements on a single low dilution li-tetraborate fused bead: JCPDS-International center for diffraction data. Adv. X-Ray Anal. 41,843–867 (1999).

[70] R. M. Conrey, D. G. Bailey, J. W. Singer, L. Wagoner, B. Parfitt, J. Hay, O. Keh, Combined Use of Multiple Internal and External Standards in LA-ICPMS Analysis of Geologic Samples Using Lithium Borate Fused Glass, poster presented at the AGU Fall Meeting, San Francisco, CA, 9 December 2019.

[71] P. Vermeesch,IsoplotR: A free and open toolbox for geochronology. Geosci. Front. 9,1479–1493 (2018).

[72] K. L. Buchan, R. Ernst, Diabase dyke swarms of Nunavut, Northwest Territories and Yukon, Canada (Open File 7464, Geological Survey of Canada, 2013).

[73] J. C. Harrison, M. R. St-Onge, O. V Petrov, S. I. Strelnikov, B. G. Lopatin, F. H. Wilson, S. Tella, D. Paul, T. Lynds, S. P. Shokalsky, C. K. Hults, S. Bergman, H. F. Jepsen, A. Solli, Geological map of the Arctic / Carte géologique de l’Arctique (Map 2159A, Geological Survey of Canada, 2011), scale 1:5,000,000.

[74] L. J. Hulbert, R. H. Rainbird, C. W. Jefferson, P. Friske, Map of Mafic and Ultramafic Bodies Related to the Franklin Magmatic Event, Minto Inlier, Victoria Island, NWT (Open File 4928, Geological Survey of Canada, 2005), scale 1:500,000.

[75] J. Hiess, D. J. Condon, N. McLean, S. R. Noble,^238^U/^235^U systematics in terrestrial uranium-bearing minerals. Science 335,1610–1614 (2012). 22461608 10.1126/science.1215507

[76] M. R. St-Onge, D. Scott, J, N. Rayner, M. Sanborn-Barrie, D. R. Skipton, B. M. Saumur, N. Wodicka, O. M. Weller, Archean and Paleoproterozoic cratonic rocks of Baffin Island, in *Geological Synthesis of Baffin Island (Nunavut) and the Labrador-Baffin Seaway*, L. T. Dafoe and N. Bingham-Koslowski, Eds. (Bulletin 608, Geological Survey of Canada, 2020), p. 29, 10.4095/321824.

[77] D. J. Condon, B. Schoene, N. M. McLean, S. A. Bowring, R. R. Parrish,Metrology and traceability of U-Pb isotope dilution geochronology (EARTHTIME tracer calibration Part I). Geochim. Cosmochim. Acta 164,464–480 (2015).

[78] T. E. Krogh,A low-contamination method for hydrothermal decomposition of zircon and extraction of U and Pb for isotopic age determinations. Geochim. Cosmochim. Acta 37,485–494 (1973).

[79] M. D. Schmitz, B. Schoene,Derivation of isotope ratios, errors, and error correlations for U-Pb geochronology using ^205^Pb-^235^U-(^233^U)-spiked isotope dilution thermal ionization mass spectrometric data. Geochem. Geophys. Geosyst. 8,1–20 (2007).

[80] J. S. Stacey, J. D. Kramers,Approximation of terrestrial lead isotope evolution by a two-stage model. Earth Planet. Sci. Lett. 26,207–221 (1975).

[81] C. Pin, J. S. Zalduegui,Sequential separation of light rare-earth elements, thorium and uranium by miniaturized extraction chromatography: Application to isotopic analyses of silicate rocks. Anal. Chim. Acta 339,79–89 (1997).

[82] R. M. Flowers, P. K. Zeitler, M. Danišík, P. W. Reiners, C. Gautheron, R. A. Ketcham, J. R. Metcalf, D. F. Stockli, E. Enkelmann, R. W. Brown,(U-Th)/He chronology: Part 1. Data, uncertainty, and reporting. GSA Bull. , (2022).

[83] R. M. Flowers, R. A. Ketcham, D. L. Shuster, K. A. Farley,Apatite (U-Th)/He thermochronometry using a radiation damage accumulation and annealing model. Geochim. Cosmochim. Acta 73,2347–2365 (2009).

[84] W. R. Guenthner, P. W. Reiners, R. A. Ketcham, L. Nasdala, G. Giester,Helium diffusion in natural zircon: Radiation damage, anisotropy, and the interpretation of zircon (U-TH)/He thermochronology. Am. J. Sci. 313,145–198 (2013).

[85] A. K. Ault, R. M. Flowers, S. A. Bowring,Phanerozoic surface history of the Slave craton. Tectonics 32,1066–1083 (2013).

[86] C. P. Sturrock, R. M. Flowers, F. A. Macdonald,The late great unconformity of the Central Canadian Shield. Geochem. Geophys. Geosyst. 22,1–22 (2021).

[87] B. A. Peak, R. M. Flowers, F. A. Macdonald, J. M. Cottle,Zircon (U-Th)/He thermochronology reveals pre-great unconformity paleotopography in the Grand Canyon region USA. Geology 49,1462–1466 (2021).

[88] R. A. Ketcham, C. Gautheron, L. Tassan-Got,Accounting for long alpha-particle stopping distances in (U-Th-Sm)/He geochronology: Refinement of the baseline case. Geochim. Cosmochim. Acta 75,7779–7791 (2011).

[89] R. A. Ketcham,Forward and inverse modeling of low-temperature thermochronometry data. Rev. Mineral. Geochem. 58,275–314 (2005).

[90] R. M. Flowers, R. A. Ketcham, E. Enkelmann, C. Gautheron, P. W. Reiners, J. R. Metcalf, M. Danišík, D. F. Stockli, R. W. Brown,(U-Th)/He chronology: Part 2. Considerations for evaluating, integrating, and interpreting conventional individual aliquot data. GSA Bull. 10.1130/B36268.1, (2022).

[91] U. Ginster, P. W. Reiners, L. Nasdala, C. Chanmuang N.,Annealing kinetics of radiation damage in zircon. Geochim. Cosmochim. Acta 249,225–246 (2019).

[92] W. R. Guenthner,Implementation of an Alpha Damage Annealing Model for Zircon (U-Th)/He Thermochronology With Comparison to a Zircon Fission Track Annealing Model. Geochem. Geophys. Geosyst. 22,e2019GC008757 (2021).

[93] R. A. Ketcham, HeFTy version 1.9.0 (2015).

[94] P. F. Hoffman, Geology and tectonics, East Arm of Great Slave Lake, Northwest Territories (Map 1628A, Geological Survey of Canada, 1988), sheet 1 of 2, scale 1:250,000 and 1:500,000.

[95] S. Polteau, A. Mazzini, O. Galland, S. Planke, A. Malthe-Sørenssen,Saucer-shaped intrusions: Occurrences, emplacement and implications. Earth Planet. Sci. Lett. 266,195–204 (2008).

[96] A. K. Ault, R. M. Flowers, S. A. Bowring,Phanerozoic burial and unroofing history of the western Slave craton and Wopmay orogen from apatite (U-Th)/He thermochronometry. Earth Planet. Sci. Lett. 284,1–11 (2009).

[97] A. K. Ault, R. M. Flowers, S. A. Bowring,Synchroneity of cratonic burial phases and gaps in the kimberlite record: Episodic magmatism or preservational bias? Earth Planet. Sci. Lett. 410,97–104 (2015).

[98] W. F. McDonough, S.-s. Sun,The composition of the Earth. Chem. Geol. 120,223–253 (1995).

[99] S.-s. Sun, W. F. McDonough,Chemical and isotopic systematics of oceanic basalts: Implications for mantle composition and processes. Geol. Soc. Spec. Publ. 42,313–345 (1989).

[100] A. H. Jaffey, K. F. Flynn, L. E. Glendenin, W. C. Bentley, A. M. Essling,Precision measurement of half-lives and specific activities of ^235^U and ^238^U. Phys. Rev. C 4,1889–1906 (1971).

[101] E. H. G. Cooperdock, R. A. Ketcham, D. F. Stockli,Resolving the effects of 2-D versus 3-D grain measurements on apatite (U-Th)/He age data and reproducibility. Geochronology 1,17–41 (2019).

[102] R. A. Wolf, K. A. Farley, D. M. Kass,Modeling of the temperature sensitivity of the apatite (U-Th)/He thermochronometer. Chem. Geol. 148,105–114 (1998).

[103] P. Martin, HeCalc (0.4.1). Zenodo (2022); 10.5281/zenodo.5672830.

