# Supplementary material for: Emplacement of the Franklin large igneous province and initiation of the Sturtian Snowball Earth
Source: Sci Adv. 2022 Nov 23;8(47):eadc9430. doi: 10.1126/sciadv.adc9430 (PMC9683727; doi:10.1126/sciadv.adc9430)
Supplement: Supplementary file 1 — Supplementary Text Figs. S1 to S5 Tables S1 to S7 References [file sciadv.adc9430_sm.pdf]

Science Advances
AAAS

Supplementary Materials for

Emplacement of the Franklin large igneous province and initiation of the Sturtian Snowball Earth

Judy P. Pu *et al.*

Corresponding author: Judy P. Pu, judypu@ucsb.edu

Sci. Adv. **8**, eadc9430 (2022)
DOI: 10.1126/sciadv.adc9430

This PDF file includes:

Supplementary Text
Figs. S1 to S5
Tables S1 to S7
References

Supplementary Text

<u>Geochemical classification of the Franklin LIP</u>

Geochemical classification of the Franklin LIP based on major and trace elements was conducted by (*36*). Further classification of Franklin LIP magmatism in the Minto Inlier was done by (*27*), splitting up Franklin LIP intrusive and volcanic rocks into northern and southern subgroups based on whether they were exposed in the northern or southern lobes of the Natkusiak Formation (Fig. 1). Northern Type 1 consists of Type 1 Franklin sills and basal basalts from the northern lobe, with low initial ε_{Nd} = -6.1 to -0.8, ε_{Hf} = -4.3 to +4.6, Nb/La = 0.42–0.67, and high initial $^{87}Sr/^{86}Sr$ = 0.7051–0.7075, consistent with some crustal contamination. Southern Type 1 consists of only basal basalts from the southern lobe, which have high ε_{Nd} = +4.4 to +8.3 and high Nb/La = 0.81–0.94 with limited evidence for crustal contamination but based on major element compositions and volcanostratigraphy still fits the older, Type 1 characterization. It was proposed by (*27*) that the composition of Southern Type 1 basalt possibly reflects a different mantle source and limited lateral mixing of the Franklin magmatic system.

Northern Type 2 consists of Type 2 Franklin sills and upper Natkusiak sheet flow basalts that have ε_{Nd} = +1 to +8.8 and ε_{Hf} = +4.7 to +15.8 (*27*), which indicate a lesser amount of continental crust contamination. Southern Type 2 includes the upper sheet flows of the southern lobe and shows higher initial ε_{Nd} = +4 to +11.8 and lower $^{87}Sr/^{86}Sr$ but otherwise similar trace element and ε_{Hf} values to Northern Type 2 (*27*).

Determining whether a sample is Type 1 or Type 2 based on major elements is less clear since major element wt. % is strongly dependent on the mineralogy of samples, and many samples in this study are more felsic than the rocks that were used to establish the geochemical trends. Previously (*36*) categorized Type 1 rocks as having low TiO_2, generally less than 1.2 wt. %. TiO_2 wt. % is similar between samples F1966 and 93JP-71M (1.80 and 1.67, respectively) and they are considered Type 2 in this framework. Sample 93JP-93L is also thought to be Type 2 and has the highest TiO_2 content at 2.98 wt. %. Sample S8 has 1.07 wt. % TiO_2 and 17RAT-R35B1 has 0.29 wt. % TiO_2, lower values for TiO_2 wt. % that are consistent with being Type 1. Sample 93JP-71JB (granitic composition), however, has relatively low wt. % TiO_2 (0.77) but otherwise reflects geochemical trends for Type 2.

<u>Sample descriptions</u>

In the Amundsen Basin, Franklin dykes and sills intruded through Archean basement granites, Paleoproterozoic sedimentary rocks of the Goulburn Supergroup, the Mesoproterozoic Coppermine River Group, and Tonian (1000–720 Ma) sedimentary rocks of the Shaler Supergroup (*28, 29*). On Baffin Island, Franklin dykes intruded the basement gneiss and metasedimentary rocks of the Archean to Paleoproterozoic Rae Craton and the Paleoproterozoic Qikiqtarjuaq plutonic suite and Cumberland batholith (*76*).

All sample coordinates and location descriptions can be found listed in Table S3. Besides F1966, all samples in this geochronology study were archived samples collected by the Geological Survey of Canada. The 93JP samples in this study were originally hand samples collected by L. Hulbert, R. Rainbird, and C. W. Jefferson in 1993. Samples 14RAT and 17RAT were collected

by R. Rainbird in 2014 and 2017. Sample FA700408 was collected by W. Fahrig in 1970. Some 93JP samples are from the same sill, denoted by their sample number, but represent different intervals and degrees of differentiation within the sill. Sample F1966 was collected on a field expedition in 2019 by F.A.M., following up on a reported Franklin age sill intruding the Great Slave Supergroup (Hearne Channel Formation, upper Pethei Group) on Great Slave Lake (*19*).

Samples in this study range from gabbroic to granitic in composition (see Table S1 for whole-rock geochemistry and Fig. S4 for representative photomicrographs of thin sections). Samples 93JP-71JB, F1966, 93JP-93L, 93JP-93K, 17RAT-R35B1, and FA700408 all show granophyric textures. Together, the samples represent six distinct sills and dykes and span a north-south distance of ~1000 km from northern Victoria Island to Great Slave Lake on mainland Canada and an east-west distance of ~2000 km from the Brock Inlier on the Amundsen Gulf coast to Cumberland Peninsula on Baffin Island (Fig. 1).

U-Pb geochronological methods

Mineral separation for the U-Pb analyses was done at Harvard University and UCSB. Since most samples were only hand samples from archive, mineral separation procedures were tailored to maximize potential yield of zircon and baddeleyite grains. All samples were hand-sledged into chips that were ≤1 cm^3 and then pulsed in 1–2 s intervals in a SPEX 8530 ShatterBox® while sieving for the <500 µm fraction. The <500 µm fraction for each sample was handwashed in 5 L beakers to remove fine material and dried under heat lamps or in low-temperature ovens before being run on the Frantz magnetic separator. Highly magnetic minerals were initially screened for and removed using a hand magnet and then samples were typically run twice, first at 0.3 A and 20° tilt and the second time at 0.6 A and 20° tilt. If less than 2 oz. of sample were left after the first run on the Frantz, the sample was not run a second time. If the sample was mostly magnetic, the tilt angle was increased by 5–10° to ensure that no non-magnetic grains were carried by the flow of magnetic grains. Heavy liquid density separation using methylene iodide was the last step for isolating the dense mineral fraction of zircon and baddeleyite. Zircon and baddeleyite grains were hand-picked for each sample from this final fraction.

Geochronological analyses were done at Boise State University. A smaller selection of zircon grains was mounted in epoxy, polished to expose grain cores, and imaged using a cathodoluminescence (CL) detector to observe igneous textures. U-Pb dates were obtained using the CA-ID-TIMS procedure developed by (*16*). Zircon and baddeleyite grains were first annealed at 900°C for 60 hours. The zircon grains were then chemically abraded using 29 M HF at 180°C or 190°C for 12 hrs. There were no major differences between analyses noted for the different chemical abrasion temperatures. The leachate was discarded, and the remaining samples were rinsed repeatedly in MQ H2O and 3.5 M HNO_3, sonicated for 30 min. and fluxed on the hot plate for 30 min. after the first round of rinsing. Samples were spiked using the EARTHTIME mixed U-Pb isotope tracer solution (ET535; *77*) and fully dissolved at 220°C for 48 hrs. U and Pb were extracted from the samples through column chemistry with AG-1 X8, 200–400 mesh, Cl^- anion exchange resin following methods modified from (*78*). Measurements were made on an IsotopX IsoProbe-T thermal ionization mass spectrometer (TIMS). Pb isotopes were measured by peak hopping on the Daly detector and U isotopes were measured in static collection mode on Faraday cups. Baddeleyite geochronology followed the same procedure except grains did not go through chemical abrasion and were instead fluxed in 3.5 M HNO_3 for

20–30 min on a hot plate and then sonicated for the same amount of time before being rinsed and loaded into microcapsules for dissolution with ET535 spike. U-Pb dates and uncertainties were calculated following (*79*). The value 137.818 ± 0.045 (2σ) was used for the $^{238}U/^{235}U$ ratio in natural zircon (*75*). Model Th/U ratios were calculated iteratively from measured $^{206}Pb/^{208}Pb$ ratios and calculated $^{206}Pb/^{238}U$ ages. Up to 1 pg of common Pb (Pbc) was assumed to be procedural blank and accounted for using the measured laboratory Pbc isotopic composition. Excess Pbc was attributed to initial common Pb using the two-stage Pb isotope evolution model (*80*) at the nominal sample age. Concordia and upper-intercept dates were plotted using IsoplotR (*71*).

Sm-Nd isotope geochemistry methods

Sm and Nd isotope geochemistry was done at the Isotope Geology Laboratory at Boise State University. A hundred mg of sample powder were spiked with a mixed ^{149}Sm-^{150}Nd tracer, dissolved with 1.8 mL 29M HF + 0.2 mL 15M HNO_3 in Parr pressure vessels at 220°C for 18 hours, dried and redissolved in 0.5 mL concentrated HNO_3 twice, then dried and redissolved in 2 mL 6M HCl at 180°C for 12 hours. Total dissolutions were dried and redissolved in 5 mL 1M HCl + 0.1M HF at 180°C overnight. Bulk rare earth elements were separated by standard dilute HCl and HNO_3 based cation exchange chemistry on 6 mm internal diameter (i.d.) x 20 cm columns of AG-50W-X8 resin, H+ form, 200–400 mesh); Sm and Nd were separated by reverse phase HDEHP chromatography on 4 mm i.d. x 10 cm columns of Eichrom Ln-spec resin, 50–100 mesh, using the methods developed by (*81*). Sm and Nd isotopes were measured on a IsotopX Phoenix X62 TIMS in static and dynamic Faraday modes, respectively. Instrumental mass fractionation of Sm and Nd isotopes was corrected with an exponential law relative to $^{146}Nd/^{144}Nd$ = 0.7219 and $^{152}Sm/^{147}Sm$ = 1.7831. $^{143}Nd/^{144}Nd$ ratio is reported as spike-stripped and bias-corrected relative to the accepted value of JNdi-1 standard (0.512106).

(U-Th)/He Thermochronology

(U-Th)/He thermochronology uses the radioactive decay of uranium (U), thorium (Th) and, to a much lesser extent, samarium (Sm) to helium (He) and the dependence of He diffusion behavior in minerals on temperature. A (U-Th)/He date represents the time-integrated history of He production and He loss over the mineral's time-temperature (t-T) history (e.g., *82*). The thermal history can be related to geological processes such as tectonic exhumation, erosion, or burial when interpreted in the context of other geologic observations such as unconformable contacts and petrological temperature indicators. In addition to temperature, He diffusion in natural materials is dependent on physical characteristics of the mineral, such as parent-nuclide zonation, grain size, and degree of crystal lattice damage from recoil during radioactive decay ("radiation damage"). Of these, radiation damage has the largest systematic effect and for some thermal histories can result in different (U-Th)/He dates among grains from the same sample. This radiation damage dependence can be used to gain information about multiple parts of the sample's thermal history that correspond to different temperatures recorded by the different sensitivities of more or less damaged grains (e.g., *83*). Radiation damage is quantified using the proxy "effective uranium" (eU) based on parent nuclide concentration as:

$$eU = [U] + 0.238[Th] + 0.0012[Sm]$$

Characteristic trends in date-eU plots can aid interpretation of datasets and can be simulated using radiation damage accumulation and annealing models for apatite (e.g., RDAAM, *83*) and zircon (e.g., ZRDAAM, *84*).

Data Collection

(U-Th)/He analysis was done at the University of Colorado Boulder Thermochronology Research and Instrumentation Lab (CU TRaIL). Individual zircon and apatite grains from sample F1966 were analyzed. The number of grains available for analysis was limited by the small size of the original sample. Zircon from other samples were unavailable for thermochronology due to annealing during geochronology data collection. AHe dates for additional samples were not obtained because apatite is more easily thermally reset than zircon, which for this region results in maximum AHe dates in the Phanerozoic (*85*)—inapplicable to the Neoproterozoic thermal history. Grains were selected using a Leica M614 binocular microscope, measured, characterized, and then packed in niobium tubes following standard procedures. Packed grains were then degassed for He measurement in an ASI Alphachron 774 extraction and measurement line. Degassed grains were dissolved and U, Th, and Sm measurements from dissolved solutions were made with an Agilent 7900 quadrupole inductively-coupled plasma mass spectrometer. Additional details of grain selection and analytical procedures for apatite and zircon are described in (*86*) and (*87*), respectively. Uncorrected dates were calculated from measured U, Th, Sm and He values and corrected for alpha-ejection based on mineral geometry following (*88*). Uncertainty on eU is conservatively assumed to be 15% of the calculated value. Date uncertainty is reported as the 2σ propagated analytical uncertainty of the U, Th, Sm, and He measurements. All data are reported in Table S6.

Inverse Thermal History Modeling and Hypothesis Testing

We carried out inverse thermal history modeling to test the consistency of the (U-Th)/He data with substantial exhumation and chemical weathering of the Franklin LIP shortly after its

emplacement due to development of a mafic volcanic highland. (U-Th)/He dates were modeled using the HeFTy software (*89*) implementing the RDAAM and the ZRDAAM. Dates were grouped together by eU and averaged, which is a commonly used approach (*90*; Table S7). The two highest eU zircon grains were excluded from modeling due to greater uncertainty in He diffusion behavior at higher damage levels (*84*, *91*, *92*). Modeling returns possible t-T paths that fit the input data within prescribed goodness of fit criteria: >0.5 for all data to be considered a "good fit" and >0.95 for all data to be considered an "acceptable fit" (*93*). We used a hypothesis-testing strategy for the two t-T simulations presented here and include key geologic constraints on the sample histories in the model frameworks (e.g., *90*).

Both models start at the time of emplacement and require surface temperatures at 515 Ma in keeping with the regional Cambrian unconformity constraint. Phanerozoic constraints on the thermal history are drawn from existing literature, particularly (*85, 94, 95*), and are derived in part from estimates of former sedimentary cover from kimberlite sedimentary xenolith suites and other geologic observations (Table S7). The Phanerozoic portion of the path is tightly constrained by the AHe data reported here, particularly from 145 Ma to the present. The differences between models are therefore in the period between emplacement and 515 Ma.

Model A was designed to test compatibility of the observed (U-Th)/He dates with the hypothesis of Franklin sill emplacement at <5 km depth followed by rapid cooling related to exhumation shortly after emplacement—the "Neoproterozoic Exhumation Hypothesis" (Fig. S5A). The starting depth of <5 km was largely based on the geometry of the Mackenzie-age Fortress Gabbro sills, which the Douglas Peninsula sill was formerly considered a part of. Although the geometry of the Douglas Peninsula sill is not fully known, the surrounding Fortress Gabbro sills have been mapped as "nested cone-sheets" based on their saucer-shape morphologies (*96*), which requires emplacement at shallow depths of 1–5 km (*97*). The model starts at 110–60°C to reflect emplacement at a typical depth (2–4 km, temperature calculated assuming an ~25°C/km geotherm and an average surface temperature of 10°C), with no constraints imposed between emplacement and surface conditions at 515 Ma. This model yields numerous good-fit paths, including those that display major cooling and erosion shortly after emplacement. This outcome indicates that the data are consistent with the hypothesis of multiple kilometers of weathering and erosion shortly after sample emplacement, possibly due, as we argue, to development and weathering of mafic volcanic highlands.

An alternative model, Model B, the "Neoproterozoic Burial/Exhumation Hypothesis," allows for a broader range of emplacement temperatures and corresponding depths from 0–6 km followed by Neoproterozoic burial prior to exhumation by 515 Ma (rather than only Neoproterozoic exhumation). We ran this model to test the uniqueness of the t-T paths generated in Model A. Similar to Model A, this simulation produces many good-fit t-T paths (Fig. S5B). This outcome indicates that the (U-Th)/He data alone do not require substantial exhumation immediately after Franklin emplacement. This can also be seen in forward model predictions using representative paths from each model simulation (Fig. S5C). However, based on the larger geologic context as described in this paper, we consider the exhumation scenario of Model A to be more geologically likely.

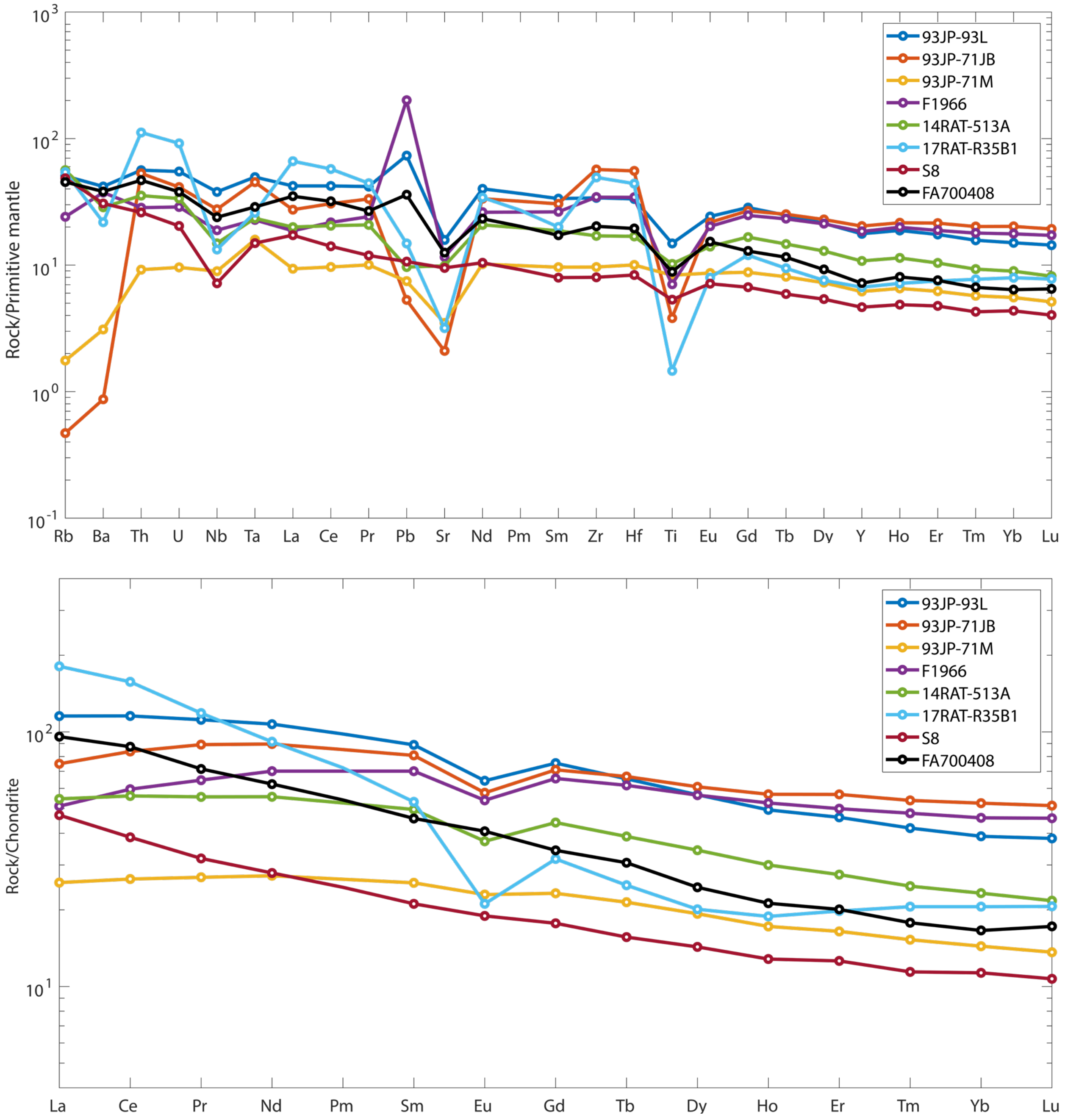

Fig. S1. Minor and trace element plots. Minor and trace elements normalized to primitive mantle (*98*) plotted above; and REE plots for each sample, normalized to chondrite (*99*) below.

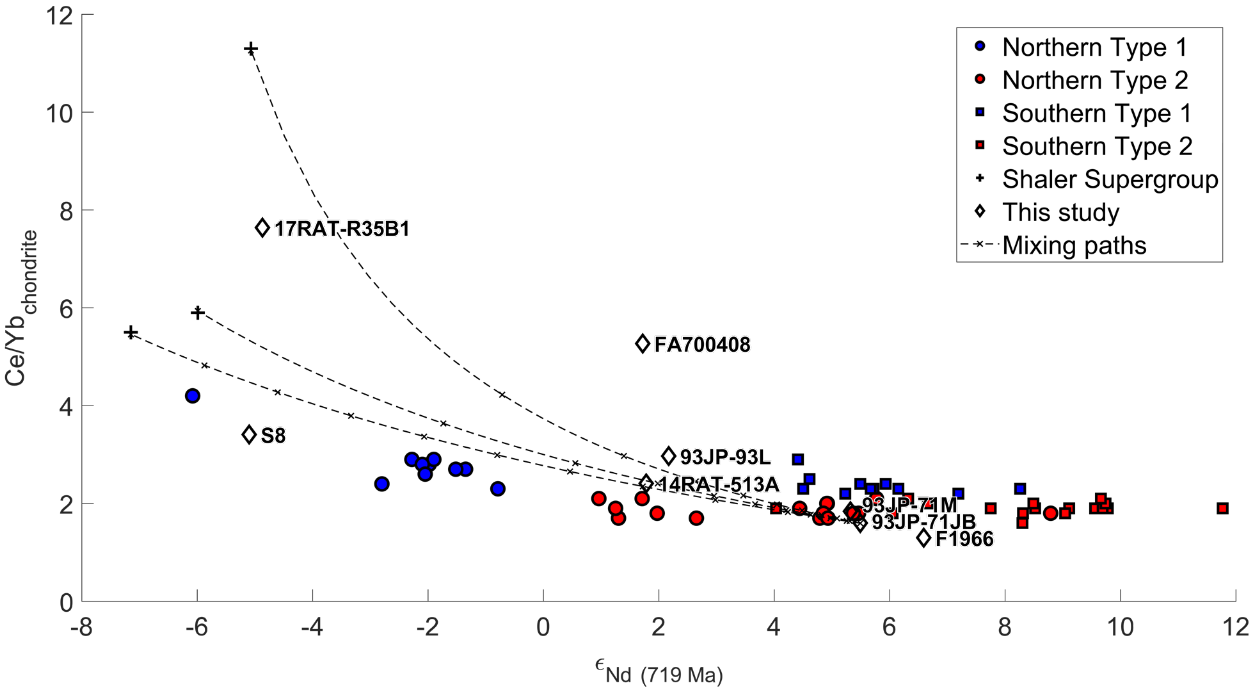

Fig. S2. (Ce/Yb)$_{chondrite}$ vs. ε_{Nd}. Values for (Ce/Yb)$_{chondrite}$ vs. ε_{Nd} calculated for 719 Ma with data from (*27*) for comparison. Most samples in this study plot similarly to Type 2 but S8 and 17RAT-R35B1 show evidence for significant crustal contamination and assimilation, more similar to Type 1 rocks. FA700408 has a positive ε_{Nd} value and does not plot close to any other group but may have a similar source to Southern Type 1 basalts with more crustal contamination. To illustrate mixing between Shaler Supergroup sedimentary rocks and Type 2 compositions in our study (represented by sample 93JP-71JB), mixing paths were calculated between the two endmembers with "x" marks denoting 10% intervals. Crustal contamination likely includes a small contribution from granitic basement rocks (not plotted) in addition to some sedimentary input (*27*).

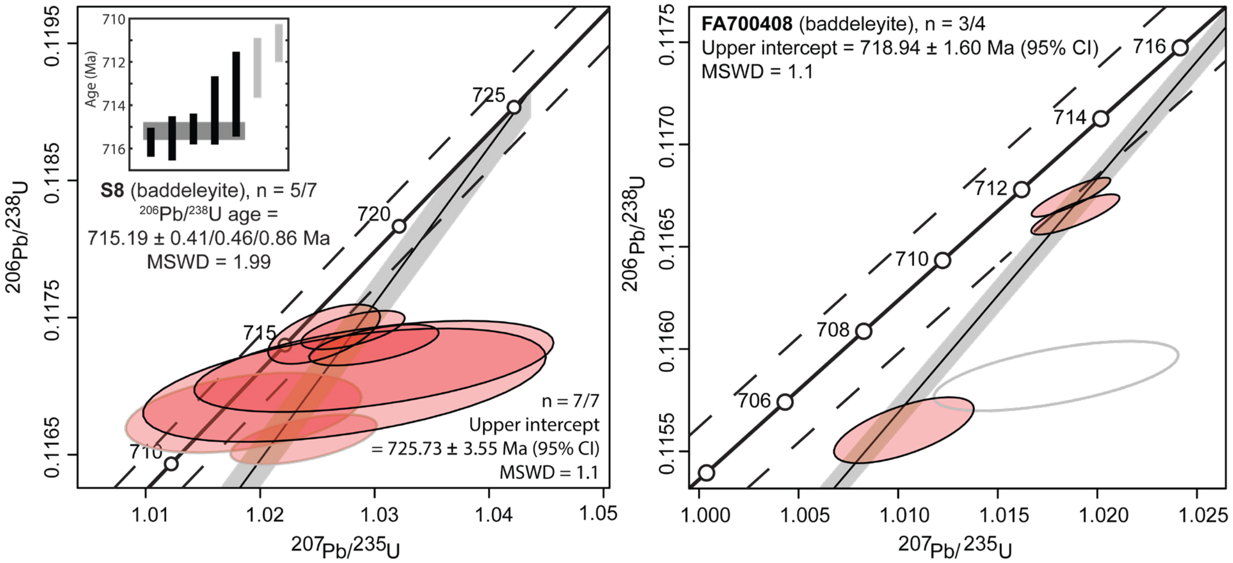

Fig. S3. Concordia plots for baddeleyite samples. Concordia plots were modified from IsoplotR (*71*). Gray data ellipses are excluded from the weighted mean dates. All analyses shown for samples S8 and FA700408 are from baddeleyite grains; all other samples only had zircon analyses (Figs. 3, 4). For S8, the two analyses with gray data ellipses were excluded from the weighted mean but included in the upper-intercept age calculation. All uncertainties are 2σ. Uncertainties for weighted mean dates in this study are reported as ± X/Y/Z, where X represents internal error only, Y includes tracer calibration uncertainties, and Z includes both tracer calibration and decay constant uncertainties for comparisons with different isotopic chronometers. The reported uncertainties for the calculated upper-intercept dates for FA700408 and S8 represent the 95% confidence interval.

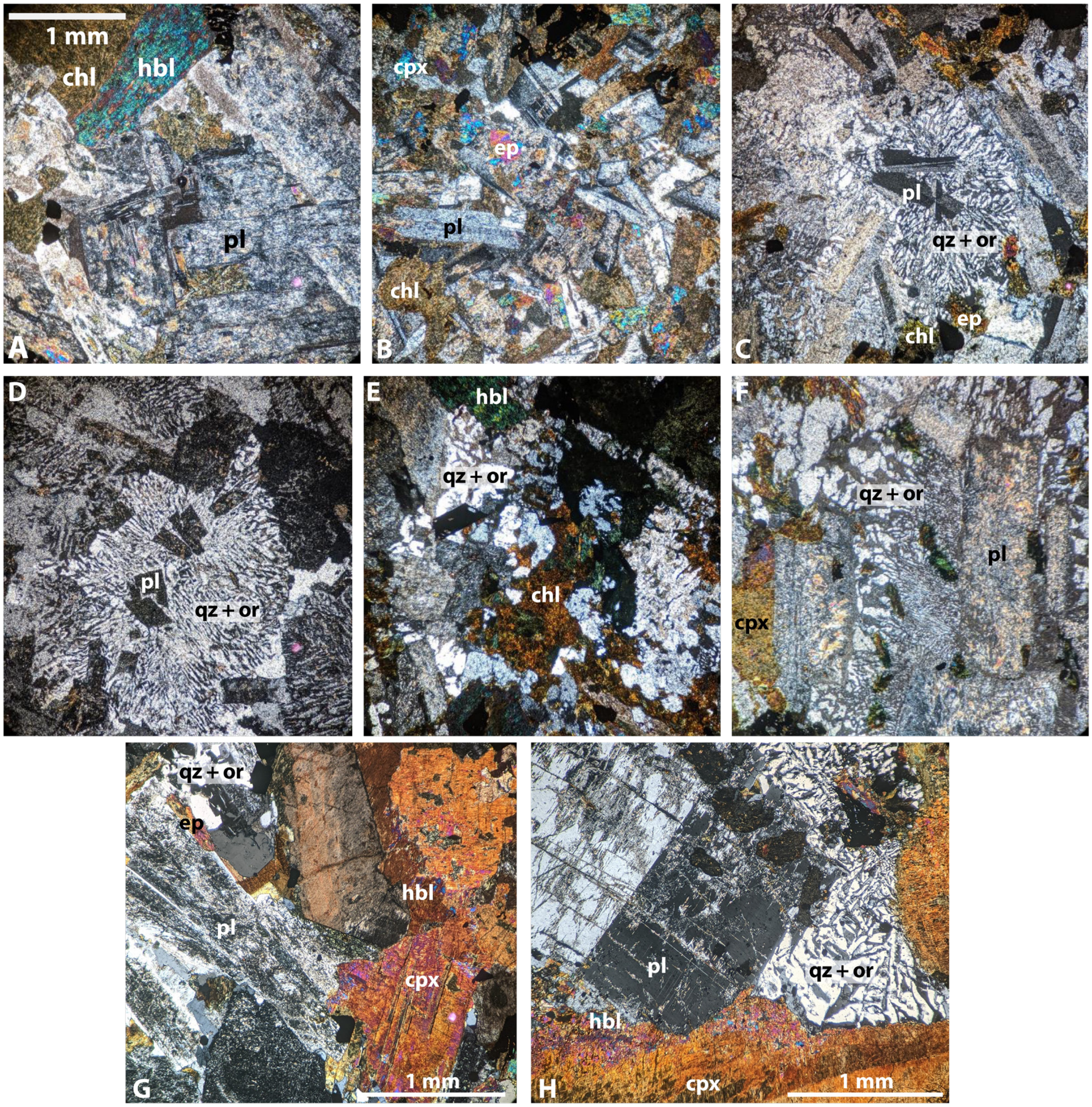

Fig. S4. Representative thin section photomicrographs. Photomicrographs of thin sections from the Franklin LIP samples in this study. Scale in upper left applies to photos **A–F**. All samples have secondary chlorite and epidote, and some also show calcite replacement. **A)** 14RAT-513A is a coarse hornblende diorite that is mostly sericite-altered plagioclase and hornblende with chlorite alteration. **B)** S8 is a gabbro. It has intergranular textures with clinopyroxene growing between plagioclase crystals. **C)** 93JP-71JB is granitic with granophyric texture enclosing plagioclase crystals and secondary epidote. Sample 71M (not shown) is from lower in the same sill and is more gabbroic in composition, with intergranular textures of plagioclase and pyroxenes. **D)** 17RAT-R35B1, granite, also has granophyric textures of K-feldspar and quartz around plagioclase crystals. **E)** 93JP-93K, hornblende quartz diorite with granophyric textures. **F)** 93JP-93L is also dioritic but more differentiated than 93JP-93K; the

sample shares a similar mineralogy to 93JP-93K but has a greater proportion of feldspars and quartz and has extensive granophyric textures. **G)** FA700408 is a gabbro composed dominantly of plagioclase feldspar and clinopyroxene with minor granophyric K-feldspar and quartz. The clinopyroxene shows alteration to hornblende. **H)** F1966 is a hornblende diorite with granophyric texture.

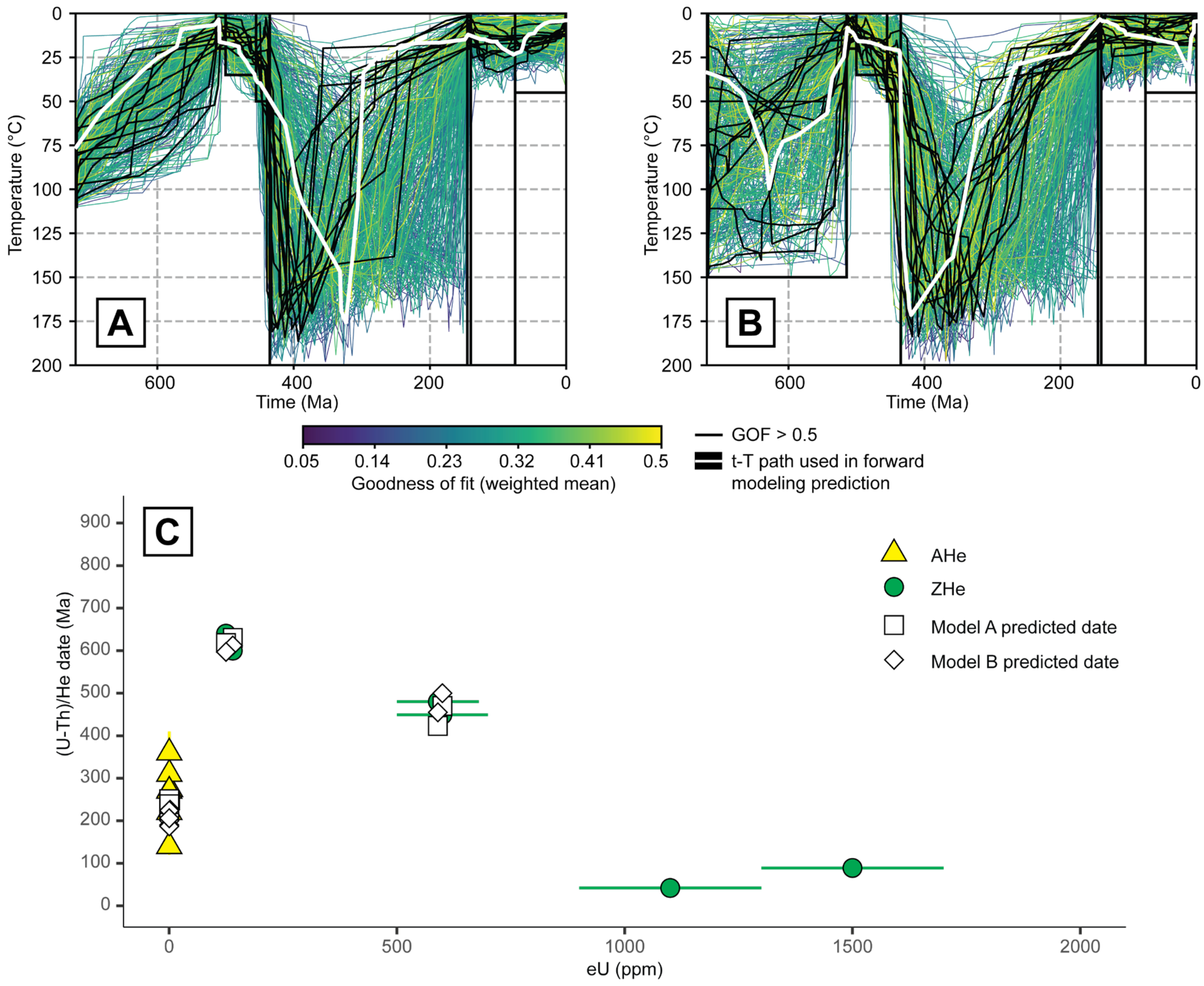

Fig. S5. U-Th/He date vs. eU and t-T model paths. Inverse thermal history modeling t-T path results for **A)** Model A: the Neoproterozoic Exhumation Hypothesis and **B)** Model B: the Neoproterozoic Burial/Exhumation Hypothesis. Color bar for acceptable fit paths is the same in all models. Black paths are good fit paths. White paths are representative good fit paths used to make predictions shown in C. Model constraints described in text and listed in Table S7 with all relevant model parameters. **C)** AHe (yellow) and ZHe (green) dates plotted against effective uranium concentration (eU). For zircon, grains with higher eU have higher levels of radiation damage and are sensitive to partial He loss at lower temperatures than lower eU grains. The two highest eU ZHe dates were excluded from modeling (see text). White symbols show forward model predictions of a representative path from each inverse model shown as white paths in A and B.

Sample	93JP-71JB	93JP-71M	93JP-93L	S8	14RAT-513A	17RAT-R35B1	FA700408	F1966
Location	Minto Inlier	Minto Inlier	Duke of York Inlier	Minto Inlier	Brock Inlier	Coppermine area	Baffin Island	Great Slave Lake
Unnormalized major element oxides (wt. %)								
SiO_2	63.86	48.35	53.48	51.43	51.28	74.77	51.22	55.44
TiO_2	0.767	1.672	2.982	1.071	2.055	0.293	2.247	1.803
Al_2O_3	11.57	13.80	11.40	14.69	15.73	9.27	14.64	10.75
$Fe_2O_3^T$	8.03	12.87	16.21	10.29	11.61	4.31	13.57	14.34
MgO	0.96	6.14	2.42	6.49	4.28	1.58	2.98	1.98
CaO	4.70	8.44	6.08	10.53	5.47	2.36	7.66	8.33
Na_2O	6.27	4.41	3.35	2.02	4.44	2.73	3.03	2.79
K_2O	0.01	0.04	1.41	1.08	1.68	0.88	1.06	2.34
P_2O_5	0.251	0.147	0.549	0.098	0.328	0.043	0.377	0.640
MnO	0.059	0.184	0.152	0.162	0.139	0.057	0.187	0.180
LOI (%)	2.29	2.78	0.90	1.11	2.45	3.39	2.00	0.47
sumMaj+LOI	98.77	98.84	98.94	98.97	99.47	99.67	98.96	99.08
sumAll	99.23	99.12	99.65	99.48	99.65	99.82	99.10	99.22
Trace elements (ppm)								
Rb	0.3	1.1	30.4	29.1	33.8	32.6	27.20	14.45
Ba	5.8	20.5	275.9	202.5	188.9	143.9	251.68	247.08
Cs	0.03	0.03	0.84	1.23	0.47	1.22	0.58	0.32
Sr	41.9	69.5	314.4	189.0	196.1	63.2	249.67	230.28
Nb	18.17	5.89	24.93	4.73	9.77	8.74	15.72	12.40
Zr	598.4	101.3	356.9	84.1	178.8	517.8	213.02	362.29
Hf	15.71	2.84	9.42	2.36	4.77	12.46	5.51	9.73
Y	87.47	26.60	75.93	20.01	46.36	28.73	31.00	79.49
Ga	24.7	20.6	25.5	17.9	23.5	12.1	21.24	23.38
Zn	42.6	61.6	62.4	58.2	57.8	14.6	189.29	37.91
Cu	30.4	243.9	389.8	168.1	9.1	16.8	32.21	77.78
Ni	5.6	64.6	3.9	68.1	24.8	6.5	11.70	<d.l.
Co							32.23	28.17
Cr	2.4	93.7	4.3	61.3	12.0	2.5	11.07	<d.l.
V	13.7	386.8	42.2	293.7	254.8	20.0	165.84	14.92
Sc	9.91	35.62	27.43	37.50	23.96	3.27	20.55	20.99
Ta	1.67	0.59	1.83	0.55	0.87	0.96	1.07	0.84
Mo	0.58	0.43	1.45	0.38	0.57	0.12	0.54	0.25
Pb	0.80	1.12	10.99	1.60	1.45	2.22	5.40	30.13
Th	4.22	0.73	4.47	2.07	2.81	8.88	3.72	2.26
U	0.84	0.19	1.11	0.41	0.68	1.86	0.77	0.58

Rare earth elements (ppm)

La	17.77	6.07	27.35	11.17	12.93	42.90	22.71	12.10
Ce	51.27	16.18	70.70	23.60	34.28	96.27	53.52	36.45
Pr	8.47	2.55	10.60	3.03	5.28	11.26	6.79	6.14
Nd	41.86	12.72	50.07	13.04	25.98	42.74	29.12	32.75
Sm	12.37	3.91	13.61	3.23	7.59	8.13	6.99	10.72
Eu	3.34	1.33	3.73	1.10	2.16	1.23	2.36	3.12
Gd	14.59	4.78	15.50	3.64	9.05	6.52	7.04	13.49
Tb	2.50	0.80	2.45	0.58	1.45	0.93	1.15	2.31
Dy	15.46	4.90	14.39	3.63	8.71	5.11	6.23	14.34
Ho	3.22	0.97	2.80	0.73	1.70	1.07	1.20	2.98
Er	9.40	2.72	7.64	2.09	4.55	3.28	3.32	8.27
Tm	1.37	0.39	1.07	0.29	0.63	0.53	0.45	1.22
Yb	8.92	2.45	6.62	1.92	3.96	3.50	2.82	7.82
Lu	1.30	0.35	0.97	0.27	0.55	0.52	0.44	1.16

Table S1. Whole rock major and trace element concentrations. Samples 93JP-71JB, 93JP-71M, 93JP-93L, S8, 14RAT-513A, and 17RAT-R35B1 were analyzed at Hamilton College. Major elements were obtained using XRF and trace elements were measured using LA-ICPMS. Samples FA700408 and F1966 were analyzed at California Institute of Technology. Major elements were measured using XRF and trace elements were measured using solution-ICPMS.

Sample	t (Ga)	[Sm] ppm	[Nd] ppm	$^{147}Sm/^{144}Nd$	± 2σ [abs]	$^{143}Nd/^{144}Nd$	± 2σ [abs]	± $2\sigma_m$	*f* Sm/Nd	Epsilon Nd (0)	Epsilon Nd (t)	t (CHUR)	t (DM)
**S8**	0.719	3.34	13.56	0.1490	0.0003	0.512152	0.000003	3	-0.2424	-9.48	-5.10	1.55	2.34
**14RAT-513A**	0.719	7.37	23.31	0.1913	0.0004	0.512703	0.000002	2	-0.0277	1.27	1.78	-1.84	3.02
**17RAT-R35B1**	0.719	8.43	42.37	0.1202	0.0002	0.512028	0.000003	3	-0.3888	-11.89	-4.87	1.21	1.83
**93JP-71JB**	0.719	12.05	40.19	0.1813	0.0004	0.512846	0.000002	2	-0.0785	4.06	5.49	-2.07	1.43
**93JP-71M**	0.719	3.87	12.80	0.1827	0.0004	0.512844	0.000002	2	-0.0713	4.02	5.32	-2.26	1.50
**93JP-93L**	0.719	13.53	48.58	0.1683	0.0003	0.512615	0.000002	2	-0.1442	-0.45	2.17	0.12	1.80
**F1966**	0.719	12.60	38.01	0.2004	0.0004	0.512993	0.000003	3	0.0190	6.92	6.59	13.87	1.81
**FA700408**	0.719	7.96	33.32	0.1444	0.0003	0.512480	0.000003	3	-0.2659	-3.09	1.72	0.46	1.47

Table S2. Sm-Nd isotope data. The quoted uncertainty for each analysis is the internal standard error; the external reproducibility of the JNdi-1 standard over the course of the study was 0.512104 ± 3 (2σ); uncertainty in [Sm], [Nd] and $^{147}Sm/^{144}Nd$ are estimated at ≤0.2% (2σ). Present-day $\varepsilon_{Nd}(0)$ and t_{CHUR} (Ga) calculated with $(^{147}Sm/^{144}Nd)_{CHUR} = 0.1967$ and $(^{143}Nd/^{144}Nd)_{CHUR} = 0.512638$; $\varepsilon_{Nd}(t)$ calculated at age of crystallization; t_{DM} (Ga) calculated with $(^{143}Nd/^{144}Nd)_{DM} = 0.513151$, $(^{147}Sm/^{144}Nd)_{DM} = 0.2137$.

Samples	Lat (degrees)	Long (degrees)	Sill/ Dyke	$^{206}Pb/^{238}U$ date (Ma)	2σ uncertainty (Ma) X	Y	Z	Notes
93JP-71JB	72.10186	-111.65593	Sill	719.04	0.19	0.28	0.79	Minto Inlier
93JP-93K	68.44681	-111.04878	Sill	718.77	0.30	0.36	0.82	Duke of York Inlier
93JP-93L	68.44681	-111.04878	Sill	718.96	0.21	0.29	0.79	Duke of York Inlier
14RAT-513A	69.63869	-120.99100	Dyke	718.61	0.30	0.36	0.82	Brock Inlier
F1966	62.72152	-110.20494	Sill	719.08	0.22	0.30	0.79	Great Slave Lake
17RAT-R35B1	67.62236	-115.48222	Sill	719.86	0.21	0.30	0.79	Coronation Gulf, Coppermine area
S8	72.18792	-111.75915	Sill	—	—	—	—	Minto Inlier
FA700408	66.52	-64.15	Dyke	—	—	—	—	Baffin Island (reversed magnetization)

Table S3. Sample locations, coordinates, and $^{206}Pb/^{238}U$ zircon dates. Uncertainties for weighted mean dates in this study are reported as ± X/Y/Z, where X represents internal error only, Y includes tracer calibration uncertainties, and Z includes both tracer calibration and decay constant uncertainties for comparisons with different isotopic chronometers.

Reference	Technique	Type of age	Mineral/Grains	Location/Formation	Rel.	Age (Ma)	(+)	(-)	max	min
(*11;* recalculated)	U-Pb ID-TIMS	upper intercept	bulk baddeleyite from six samples	Coronation sills, Quadyuk Island gabbro, and Cumberland dyke	sills and dykes	724	3	3	727	721
(*11;* recalculated)	U-Pb ID-TIMS	upper intercept	bulk baddeleyite from three sills	Victoria Island (Upper and Middle Sills)	sills	723	1	1	724	722
(*13*)	U-Pb ID-TIMS	$^{206}Pb/^{238}U$ weighted mean	1 & 2 grain baddeleyite b.f.	Qaanaaq dyke	dyke	721	4	4	725	717
(*13*)	U-Pb ID-TIMS	$^{206}Pb/^{238}U$ weighted mean	1 & 2 grain baddeleyite b.f.	Cadogan Glacier dyke	dyke	721	2	2	723	719
(*12*)	U-Pb ID-TIMS	$^{206}Pb/^{238}U$ weighted mean	bulk baddeleyite	Borden dykes	dyke	720	8	8	728	712
17RAT-R35B1 (this study)	U-Pb CA-ID-TIMS	$^{206}Pb/^{238}U$ weighted mean	single grain zircon	Coppermine area	sill	719.86	0.30	0.30	720.16	719.56
F1966 (this study)	U-Pb CA-ID-TIMS	$^{206}Pb/^{238}U$ weighted mean	single grain zircon	Great Slave Lake	sill	719.08	0.30	0.30	719.38	718.78
93JP71JB (this study)	U-Pb CA-ID-TIMS	$^{206}Pb/^{238}U$ weighted mean	single grain zircon	Minto Inlier	sill	719.04	0.28	0.28	719.32	718.76
93JP93K (this study)	U-Pb CA-ID-TIMS	$^{206}Pb/^{238}U$ weighted mean	single grain zircon	Duke of York Inlier	sill	718.77	0.36	0.36	719.13	718.41
93JP93L (this study)	U-Pb CA-ID-TIMS	$^{206}Pb/^{238}U$ weighted mean	single grain zircon	Duke of York Inlier	sill	718.96	0.29	0.29	719.25	718.67
14RAT-513A (this study)	U-Pb CA-ID-TIMS	$^{206}Pb/^{238}U$ weighted mean	single grain zircon	Brock Inlier	dyke	718.61	0.36	0.36	718.97	718.25
FA700408 (this study)	U-Pb ID-TIMS	upper intercept	single grain baddeleyite	Baffin Island	dyke	718.94	1.60	1.60	720.54	717.34
(*11;* recalculated)	U-Pb ID-TIMS	upper intercept	bulk zircon and baddeleyite	Victoria Island (Lower sill)	sills	718	3	3	721	715
(*19*)	U-Pb CA-ID-TIMS	concordia age	single grain zircon and baddeleyite	Great Slave Lake	sill	716.2	1.9	1.9	718.1	714.3
(*10*)	U-Pb ID-TIMS	$^{206}Pb/^{238}U$ weighted mean	single grain baddeleyite	Clarence head	dyke	716	1	1	717	715
(*12*)	U-Pb ID-TIMS	$^{206}Pb/^{238}U$ weighted mean	bulk baddeleyite	Borden dykes	dyke	716	4	5	720	711
(*4;* (recalculated)	U-Pb ID-TIMS	$^{206}Pb/^{238}U$ weighted mean	single grain baddeleyite	Sill 6a, Shaler Gp	sill	715.19	0.46	0.46	715.65	714.73
(*4;* (recalculated)	U-Pb ID-TIMS	upper intercept	single grain baddeleyite	Sill 6a, Shaler Gp	sill	725.73	3.55	3.55	729.28	722.18
(*10*)	U-Pb ID-TIMS	$^{206}Pb/^{238}U$ weighted mean	single grain baddeleyite	Clarence head	dyke	713	2	2	715	711
(*10*)	U-Pb ID-TIMS	$^{206}Pb/^{238}U$ weighted mean	bulk baddeleyite	Clarence head	dyke	713	3	3	716	710
(*13*)	U-Pb ID-TIMS	$^{206}Pb/^{238}U$ weighted mean	bulk baddeleyite	Granville Fjord sill	sill	712	2	2	714	710

Table S4. Existing geochronological constraints on the Franklin LIP. Previously published upper-intercept and weighted mean ages for the Franklin LIP along with the results from this study. Uncertainties for weighted mean ages from this study incorporate both

internal errors and external errors associated with tracer calibrations for interlaboratory comparisons of U-Pb analyses. Uncertainties for previously published ages include tracer calibration error in addition to internal analytical error where available. Upper-intercept ages are reported with 95% CI uncertainties and are recalculated from the literature using $^{238}U/^{235}U$ = 137.818 (*75*) in IsoplotR (*71*).

	Compositional Parameters					Radiogenic Isotope Ratios							Isotopic Ages					
Sample	Th/U	Pb*/Pbc	Pbc (pg)	206Pb/204Pb	208Pb/206Pb	207Pb/206Pb	2σ % err	207Pb/235U	2σ % err	206Pb/238U	2σ % err	corr. coef.	207Pb/206Pb	2σ abs	207Pb/235U	2σ abs	206Pb/238U	2σ abs
(a)	(b)	(c)	(c)	(d)	(e)	(e)	(f)	(e)	(f)	(e)	(f)		(g)	(f)	(g)	(f)	(g)	(f)
**93JP-71JB**																		
z1	2.034	228	0.22	9773	0.629	0.063347	0.073	1.030515	0.133	0.118038	0.066	0.960	718.94	1.54	719.18	0.69	719.26	0.45
z2	2.055	17	0.47	729	0.635	0.063137	0.453	1.026479	0.508	0.117967	0.108	0.590	711.88	9.63	717.16	2.61	718.85	0.73
z3	2.189	20	0.57	865	0.677	0.063111	0.353	1.025959	0.402	0.117956	0.087	0.630	711.02	7.51	716.90	2.07	718.79	0.60
z4	1.942	7	0.50	305	0.600	0.063328	1.093	1.029066	1.240	0.117907	0.413	0.502	718.31	23.20	718.46	6.39	718.51	2.81
z5	2.942	149	0.26	5486	0.910	0.063499	0.090	1.032973	0.162	0.118037	0.095	0.883	724.03	1.91	720.41	0.84	719.25	0.65
z6	2.497	2	0.41	114	0.772	0.063677	4.628	1.034103	4.809	0.117835	0.523	0.395	729.97	98.06	720.98	24.82	718.09	3.55
z7	3.683	10	0.33	354	1.139	0.063036	1.831	1.022544	2.033	0.117703	0.604	0.466	708.50	38.94	715.19	10.44	717.33	4.10
z8	2.834	7	0.33	265	0.876	0.063233	1.482	1.027992	1.616	0.117961	0.357	0.471	715.13	31.48	717.92	8.32	718.82	2.43
z9	2.387	7	4.36	301	0.738	0.063448	0.387	1.032033	0.435	0.118023	0.105	0.550	722.34	8.21	719.94	2.24	719.17	0.71
z10	3.585	82	1.21	2716	1.108	0.063412	0.096	1.030891	0.153	0.117960	0.067	0.911	721.14	2.04	719.37	0.79	718.81	0.45
z11	2.204	13	3.78	558	0.681	0.063374	0.214	1.030687	0.264	0.118007	0.084	0.700	719.86	4.54	719.27	1.36	719.08	0.57
z12	2.524	10	3.62	397	0.780	0.063633	0.279	1.034792	0.330	0.117995	0.099	0.621	728.51	5.92	721.32	1.70	719.01	0.67
z13	3.431	347	0.46	11840	1.061	0.063389	0.068	1.030914	0.132	0.118006	0.071	0.953	720.35	1.44	719.38	0.68	719.08	0.48
**93JP-71M**																		
z1	2.700	15	0.26	599	0.835	0.063093	0.610	1.024895	0.733	0.117867	0.323	0.569	710.40	12.97	716.37	3.77	718.28	2.20
**93JP-93K**																		
z1	1.414	20	0.22	962	0.437	0.063342	0.450	1.029251	0.512	0.117902	0.135	0.563	718.79	9.56	718.55	2.64	718.48	0.92
z2a	1.525	43	0.29	2048	0.472	0.063410	0.196	1.019995	0.244	0.116717	0.082	0.703	721.06	4.16	713.91	1.25	711.64	0.55
z2b	1.432	36	0.87	1734	0.443	0.063502	0.143	1.028599	0.193	0.117531	0.069	0.816	724.14	3.02	718.23	0.99	716.33	0.47
z3	0.804	5	0.73	266	0.249	0.063531	0.921	1.031102	1.000	0.117762	0.149	0.587	725.12	19.53	719.48	5.16	717.67	1.01
z4	0.742	10	0.20	577	0.229	0.063486	0.831	1.030876	0.901	0.117822	0.170	0.491	723.59	17.63	719.37	4.64	718.01	1.16
z5	0.526	29	0.64	1749	0.163	0.063403	0.175	1.024492	0.232	0.117245	0.098	0.721	720.81	3.71	716.17	1.19	714.69	0.66
z6a	0.940	61	0.27	3284	0.291	0.063478	0.115	1.028800	0.169	0.117599	0.069	0.865	723.33	2.44	718.33	0.87	716.73	0.47
z6b	0.795	33	0.50	1831	0.246	0.063396	0.200	1.011702	0.247	0.115794	0.081	0.695	720.58	4.25	709.73	1.26	706.31	0.54
z7a	0.989	106	0.57	5624	0.306	0.063475	0.086	1.032018	0.142	0.117972	0.066	0.919	723.23	1.83	719.94	0.73	718.88	0.45
z7b	1.034	134	0.19	7067	0.320	0.063356	0.089	1.030049	0.146	0.117968	0.069	0.897	719.24	1.90	718.95	0.75	718.86	0.47
**93JP-93L**																		

z1	1.304	50	0.67	2473	0.403	0.063421	0.126	1.031223	0.182	0.117982	0.080	0.815	721.41	2.67	719.54	0.94	718.94	0.54
z2	2.749	3	1.45	121	0.850	0.062439	1.961	1.012460	2.092	0.117657	0.267	0.538	688.21	41.84	710.12	10.69	717.07	1.81
z3	1.313	10	0.82	503	0.406	0.063415	0.441	1.030067	0.502	0.117860	0.131	0.568	721.23	9.35	718.96	2.58	718.23	0.89
z4	1.520	67	0.67	3181	0.470	0.063435	0.106	1.032061	0.160	0.118051	0.068	0.874	721.90	2.24	719.96	0.82	719.33	0.46
z5	1.356	376	0.34	18392	0.419	0.063418	0.065	1.031196	0.128	0.117984	0.069	0.961	721.32	1.39	719.53	0.66	718.95	0.47
z7	1.335	11	0.34	561	0.413	0.063394	0.610	1.031570	0.672	0.118071	0.129	0.553	720.52	12.94	719.71	3.46	719.45	0.88
z8	1.192	24	0.81	1233	0.368	0.063404	0.193	1.031102	0.252	0.118000	0.104	0.708	720.85	4.09	719.48	1.30	719.04	0.71
z9	2.042	7	0.66	318	0.631	0.063538	0.781	1.032277	0.854	0.117885	0.138	0.584	725.33	16.57	720.07	4.41	718.38	0.94
z10	1.387	5	2.50	263	0.429	0.063596	0.436	1.032322	0.500	0.117782	0.165	0.528	727.27	9.25	720.09	2.58	717.79	1.12
z13	1.500	36	1.01	1721	0.464	0.063327	0.141	1.030307	0.206	0.118052	0.099	0.789	718.26	3.00	719.08	1.06	719.34	0.68
z14	1.670	125	0.18	5740	0.516	0.063380	0.099	1.026917	0.178	0.117564	0.114	0.860	720.06	2.10	717.38	0.92	716.53	0.77
z15	1.146	63	0.16	3251	0.354	0.063306	0.146	1.024740	0.236	0.117452	0.107	0.910	717.58	3.10	716.29	1.22	715.88	0.73
z16	1.939	23	0.22	1012	0.599	0.063297	0.394	1.029157	0.446	0.117976	0.117	0.551	717.27	8.37	718.51	2.30	718.90	0.79

17RAT-R35B1

z1	1.235	18	1.27	926	0.382	0.063260	0.210	1.027036	0.268	0.117801	0.110	0.677	716.03	4.45	717.44	1.38	717.90	0.75
z2	1.001	7	2.29	405	0.310	0.063217	0.397	1.026851	0.451	0.117861	0.111	0.580	714.57	8.43	717.35	2.32	718.24	0.76
z3	0.956	21	1.09	1138	0.296	0.063536	0.165	1.034765	0.219	0.118173	0.080	0.769	725.26	3.51	721.31	1.13	720.04	0.55
z4	1.066	48	0.44	2513	0.330	0.063399	0.160	1.037021	0.208	0.118687	0.076	0.737	720.67	3.40	722.43	1.07	723.00	0.52
z5	1.148	32	2.34	1662	0.355	0.063480	0.105	1.033908	0.166	0.118179	0.076	0.880	723.39	2.24	720.88	0.86	720.08	0.52
z6	0.961	7	1.22	380	0.297	0.063378	0.413	1.029010	0.468	0.117809	0.104	0.608	719.97	8.76	718.43	2.41	717.94	0.71
z7	1.171	58	0.49	2947	0.362	0.063404	0.126	1.031909	0.177	0.118091	0.073	0.813	720.87	2.66	719.88	0.91	719.57	0.50
z8	0.988	27	0.48	1466	0.305	0.063521	0.224	1.033142	0.270	0.118015	0.077	0.691	724.77	4.74	720.50	1.39	719.13	0.52
z10	1.000	22	1.38	1157	0.309	0.063265	0.165	1.029609	0.221	0.118087	0.090	0.750	716.20	3.50	718.73	1.14	719.54	0.61
z11	1.112	44	0.75	2251	0.344	0.063227	0.133	1.028056	0.194	0.117980	0.092	0.795	714.92	2.83	717.95	1.00	718.93	0.63
z12	1.298	60	1.85	2945	0.401	0.063400	0.085	1.031136	0.146	0.118010	0.068	0.940	720.72	1.81	719.50	0.75	719.10	0.46
z13	1.129	44	0.70	2249	0.349	0.063415	0.138	1.033342	0.188	0.118234	0.072	0.792	721.24	2.93	720.60	0.97	720.39	0.49
z14	1.528	89	0.35	4222	0.473	0.063418	0.109	1.029311	0.166	0.117768	0.080	0.832	721.33	2.31	718.58	0.85	717.70	0.54
z15	1.350	80	0.55	3919	0.417	0.063441	0.101	1.031242	0.158	0.117947	0.073	0.868	722.09	2.14	719.55	0.81	718.73	0.50
z16	1.383	22	2.03	1101	0.428	0.063442	0.138	1.032344	0.194	0.118071	0.075	0.831	722.13	2.93	720.10	1.00	719.45	0.51

14RAT-513A

z1	1.834	12	0.87	543	0.567	0.063544	0.367	1.031804	0.427	0.117820	0.123	0.598	725.53	7.78	719.83	2.20	718.00	0.84
z2	3.805	3	7.34	117	1.176	0.063701	1.037	1.037117	1.113	0.118135	0.332	0.370	730.76	21.96	722.48	5.75	719.82	2.26
z3	1.100	3	0.68	166	0.340	0.062943	2.340	1.020348	2.511	0.117624	0.339	0.555	705.34	49.78	714.09	12.87	716.87	2.30

z5	1.356	28	0.37	1385	0.419	0.063396	0.269	1.026548	0.344	0.117492	0.160	0.647	720.60	5.72	717.20	1.77	716.11	1.08
z6	2.208	26	0.93	1090	0.683	0.063300	0.226	1.027946	0.281	0.117831	0.099	0.674	717.38	4.80	717.90	1.44	718.07	0.67
z8	2.516	20	1.53	794	0.778	0.063607	0.288	1.033127	0.404	0.117853	0.242	0.711	727.65	6.10	720.49	2.09	718.19	1.65
z13	4.240	273	0.31	8337	1.311	0.063459	0.096	1.032554	0.149	0.118063	0.070	0.861	722.69	2.04	720.20	0.77	719.41	0.47
z14	3.065	130	0.17	4685	0.948	0.063458	0.109	1.029476	0.230	0.117714	0.178	0.889	722.65	2.30	718.67	1.18	717.39	1.21
z15	1.725	62	0.17	2814	0.533	0.063479	0.193	1.034470	0.371	0.118244	0.295	0.856	723.37	4.10	721.16	1.91	720.45	2.01
z17	2.715	36	0.34	1374	0.839	0.063255	0.264	1.026981	0.326	0.117804	0.131	0.629	715.87	5.62	717.42	1.68	717.91	0.89
**F1966**																		
z1	0.968	152	0.23	8108	0.299	0.063442	0.079	1.030571	0.148	0.117869	0.085	0.908	722.11	1.68	719.21	0.76	718.28	0.58
z2	2.416	19	0.41	786	0.747	0.063181	0.496	1.023119	0.551	0.117499	0.114	0.560	713.37	10.54	715.48	2.83	716.15	0.77
z3	1.858	11	0.39	523	0.575	0.063330	0.707	1.026128	0.779	0.117567	0.137	0.593	718.38	15.01	716.99	4.01	716.55	0.93
z4	3.537	105	0.27	3527	1.094	0.063371	0.121	1.029234	0.175	0.117846	0.078	0.811	719.76	2.57	718.54	0.90	718.16	0.53
z5	3.200	230	0.24	8123	0.989	0.063364	0.080	1.030062	0.138	0.117954	0.066	0.933	719.53	1.70	718.96	0.71	718.78	0.45
z6	0.953	103	0.35	5507	0.295	0.063354	0.093	1.028290	0.148	0.117770	0.066	0.900	719.19	1.97	718.07	0.76	717.72	0.45
z7	0.709	18	1.88	1008	0.219	0.063511	0.156	1.032828	0.208	0.117998	0.074	0.799	724.42	3.30	720.34	1.08	719.03	0.51
z8	2.931	34	2.95	1268	0.906	0.063368	0.118	1.034698	0.174	0.118479	0.069	0.879	719.64	2.51	721.27	0.90	721.80	0.47
z9	2.537	45	0.76	1793	0.784	0.063416	0.157	1.028742	0.222	0.117708	0.110	0.752	721.24	3.34	718.30	1.14	717.36	0.75
z10	3.027	427	0.38	15460	0.936	0.063379	0.068	1.031043	0.128	0.118039	0.065	0.965	720.03	1.43	719.45	0.66	719.26	0.44
z11	2.989	66	1.16	2388	0.924	0.063400	0.082	1.031547	0.147	0.118057	0.066	0.985	720.74	1.75	719.70	0.76	719.37	0.45
z12	0.892	15	0.64	805	0.276	0.063505	0.333	1.033740	0.416	0.118112	0.186	0.625	724.25	7.06	720.80	2.15	719.69	1.27
z13	1.284	74	0.46	3706	0.397	0.063366	0.122	1.029005	0.169	0.117830	0.068	0.802	719.59	2.59	718.43	0.87	718.06	0.46
z14	2.299	145	0.37	5927	0.711	0.063341	0.086	1.033956	0.142	0.118443	0.066	0.921	718.76	1.82	720.90	0.73	721.59	0.45
z15	2.234	57	0.35	2376	0.691	0.063367	0.155	1.032265	0.207	0.118201	0.078	0.773	719.62	3.30	720.06	1.07	720.20	0.53
z16	2.971	122	0.31	4475	0.919	0.063401	0.101	1.029740	0.156	0.117848	0.069	0.874	720.77	2.14	718.80	0.80	718.17	0.47
z17	2.214	37	0.36	1565	0.685	0.063542	0.306	1.032639	0.355	0.117919	0.107	0.578	725.46	6.49	720.25	1.83	718.57	0.73
z18	3.606	58	0.34	1951	1.115	0.063465	0.192	1.028951	0.246	0.117641	0.096	0.696	722.88	4.07	718.40	1.27	716.97	0.65
**FA700408**																		
b1	0.080	25	0.54	1684	0.025	0.063412	0.227	1.010288	0.284	0.115603	0.114	0.649	721.11	4.83	709.02	1.45	705.21	0.76
b2	0.396	11	1.16	689	0.122	0.063745	0.431	1.017929	0.493	0.115868	0.120	0.602	732.24	9.14	712.87	2.52	706.74	0.80
b3	0.155	45	0.52	2955	0.048	0.063374	0.127	1.018895	0.178	0.116658	0.069	0.821	719.84	2.70	713.36	0.91	711.30	0.46
b4	0.246	66	0.51	4251	0.076	0.063315	0.106	1.018676	0.159	0.116742	0.067	0.870	717.86	2.25	713.25	0.81	711.78	0.45
z1	0.938	3	0.55	168	0.290	0.063750	1.964	1.020122	2.119	0.116109	0.402	0.467	732.39	41.59	713.97	10.87	708.13	2.70

**S8**																		
b1	0.078	10	0.50	708	0.024	0.063704	0.451	1.023801	0.510	0.116611	0.128	0.563	730.87	9.55	715.82	2.62	711.03	0.86
b2	0.097	16	0.41	1080	0.030	0.063537	0.309	1.028104	0.360	0.117409	0.098	0.617	725.32	6.55	717.98	1.85	715.63	0.66
b4	0.057	12	0.46	855	0.018	0.063710	0.396	1.029943	0.449	0.117300	0.104	0.597	731.06	8.38	718.90	2.31	715.01	0.71
b5	0.128	4	1.03	267	0.039	0.063794	1.143	1.029931	1.246	0.117144	0.232	0.518	733.86	24.21	718.89	6.42	714.11	1.57
b6	0.088	16	0.46	1058	0.027	0.063398	0.324	1.025582	0.390	0.117379	0.151	0.591	720.65	6.88	716.72	2.00	715.46	1.02
b7	0.181	9	0.50	612	0.056	0.063272	0.770	1.018552	0.826	0.116806	0.204	0.389	716.44	16.36	713.19	4.23	712.15	1.37
b8	0.089	3	0.85	251	0.028	0.063710	1.281	1.027362	1.400	0.117007	0.289	0.495	731.05	27.15	717.61	7.20	713.31	1.95

Table S5. U-Pb data table for all samples. Analyses in red were not included in calculations for mean sample dates. Analyses that were not run to completion due to high Pb blanks and/or low amounts of radiogenic Pb have been removed. The value 137.818 ± 0.045 (2σ) was used for the $^{238}U/^{235}U$ ratio in natural zircon (*75*). (a) z1, z2, etc. and b1, b2, etc. are labels for single zircon or baddeleyite grains/fragments, respectively. Zircon grains were annealed and chemically abraded after (*16*); (b) Model Th/U ratio iteratively calculated from the radiogenic $^{208}Pb/^{206}Pb$ ratio and $^{206}Pb/^{238}U$ age; (c) Pb* and Pbc represent radiogenic and common Pb, respectively; mol % $^{206}Pb^*$ with respect to radiogenic, blank and initial common Pb; (d) Measured ratio corrected for spike and fractionation only. Fractionation estimated at 0.18 ± 0.03 %/a.m.u. for Daly analyses, based on analysis of NBS-981 and NBS-982; (e) Corrected for fractionation, spike, and common Pb; up to 1 pg of common Pb was assumed to be procedural blank: $^{206}Pb/^{204}Pb$ = 18.042 ± 0.61%; $^{207}Pb/^{204}Pb$ = 15.537 ± 0.52%; $^{208}Pb/^{204}Pb$ = 37.686 ± 0.63% (all uncertainties 1σ). Excess over blank was assigned to initial common Pb, using the (*80*) two-stage Pb isotope evolution model at the nominal sample age; (f) Errors are 2σ, propagated using the algorithms of (*79*); (g) Calculations are based on the decay constants of (*100*). $^{206}Pb/^{238}U$ and $^{207}Pb/^{206}Pb$ ages corrected for initial disequilibrium in $^{230}Th/^{238}U$ using Th/U [magma] = 3; (h) Corrected for fractionation, spike, and blank Pb only.

Sample Name and aliquot[a,b]	length 1 (µm)[c]	width 1 (µm)[d]	length 2 (µm)[c]	width 2 (µm)[d]	Geometry[e]	Np[f]	^{4}He (fmol)[g]	±[h]	U (ng)[i]	±[h]	Th (ng)[j]	±[h]	^{147}Sm (ng)[k]	±[h]	Rs (µm)[l]	Mass (µg)[m]
F1966																
a01	220	88	220	69	4	1	5.40	0.06	0.0030	0.0002	0.0069	0.0003	0.0783	0.0022	42	2.4
a02	200	123	349	78	4	0	5.77	0.08	0.0025	0.0003	0.0056	0.0003	0.0725	0.0017	60	6.9
a03	311	100	321	94	4	0	3.17	0.03	0.0014	0.0002	0.0075	0.0003	0.0484	0.0017	58	6.9
a04	372	94	380	91	4	1	4.99	0.05	0.0019	0.0002	0.0039	0.0003	0.0546	0.0017	57	6.8
a05	339	157	345	153	4	1	9.21	0.08	0.0049	0.0005	0.0078	0.0003	0.0919	0.0013	87	15.9
a06	451	110	444	107	4	1	2.91	0.03	0.0033	0.0004	0.0047	0.0002	0.0566	0.0015	67	11.2
z01	578	373	495	284	3	0	119000	300	29.9	0.5	31.6	0.6	n.m	n.m	186	265
z02	524	341	539	293	3	0	105000	300	25.1	0.6	24.0	0.3	n.m	n.m	182	246
z03	111	68	108	63	3	2	1397	13	0.632	0.017	0.556	0.019	n.m	n.m	38	1.3
z04	123	51	123	48	3	2	464	3	0.92	0.03	2.6	0.6	n.m	n.m	33	1.0
z05	155	78	156	70	3	2	495	3	1.82	0.03	4.8	0.5	n.m	n.m	46	2.7
z06	185	96	186	67	3	1	5790	30	2.19	0.04	3.59	0.06	n.m	n.m	51	4.7

Sample Name and aliquot[a,b]	^{4}He (nmol/g)[n]	±[o]	U (ppm)[n]	±[o]	Th (ppm)[n]	±[o]	Sm (ppm)[n]	±[o]	eU (ppm)[p]	±[q]	Uncorr Date (Ma)[r]	Uncorr Date Analytical ± (Ma) 2σ[s]	F_T comb[t]	Corrected Date (Ma)[u]	± TAU (Ma) 2σ[v]
F1966															
a01	2.24	0.05	1.2	0.2	2.9	0.3	217	12	2.2	0.3	188	15	0.65	274	22
a02	0.84	0.02	0.36	0.09	0.82	0.08	70	3	0.64	0.10	240	30	0.75	310	50
a03	0.46	0.01	0.20	0.06	1.09	0.10	47	3	0.51	0.08	160	20	0.74	220	30
a04	0.73	0.02	0.28	0.06	0.57	0.10	53	3	0.48	0.07	280	40	0.74	360	50
a05	0.58	0.01	0.31	0.07	0.49	0.04	39	1	0.47	0.07	220	30	0.83	270	40
a06	0.26	0.01	0.29	0.07	0.42	0.04	34	2	0.43	0.07	110	17	0.78	140	20
z01	450	2	113	4	120	4	n.m	n.m	140	20	564	15	0.94	600	16
z02	426	2	102	5	97	2	n.m	n.m	125	19	600	20	0.93	640	20
z03	1070	20	480	30	430	30	n.m	n.m	590	90	330	16	0.69	480	20
z04	454	6	900	50	2600	1100	n.m	n.m	1500	200	60	10	0.63	89	16
z05	185	2	680	30	1800	400	n.m	n.m	1100	200	31	3	0.73	42	4
z06	1232	13	466	17	770	20	n.m	n.m	600	100	340	10	0.76	449	13

Table S6. Apatite and zircon (U-Th)/He data. (a) This table follows the recommendations and approach in (*82*). (b) Sample and mineral being analyzed. a is apatite. z is zircon. (c) Length is measured parallel to the c-axis and includes pyramidal terminations. It is measured twice on two perpendicular sides. (d) Width 1 is measured perpendicular to the c-axis. Width 2 is measured perpendicular to both the c-axis and width 1. (e) Geometry is defined as described as in Figure 3 of (*88*). 1 is ellipsoid, 2 is cylinder, 3 is tetrahedral prism, and 4 is hexagonal prism. f is noted if the analyzed grain is a fragment, otherwise the analyzed grain is a whole crystal. (f) Np denotes the number of pyramidal terminations of the grain. (g) Blank-corrected ^{4}He. (h) Uncertainties on ^{4}He, U, Th, and Sm are reported as the 1σ standard deviation and include the propagated uncertainties on the measurements of the sample, blank, spike, and standard. (i) Total blank-corrected ng of ^{238}U and ^{235}U. Total ^{238}U is measured and ^{235}U is calculated assuming $^{235}U = {}^{238}U/137.818$ after (*75*). (j) Total blank-corrected ng of ^{232}Th. (k) Total blank-corrected ng of ^{147}Sm. In some cases, Sm may not be measured, for example in minerals like zircon with negligible Sm. "n.m." indicates when Sm is not measured. (l) Rs is the radius of a sphere with an equivalent alpha-ejection correction as the grain, calculated using equation A6 in (*101*). (m) Mass is the mass of the crystal. Determined from the measured grain dimensions, the volume assuming the reported grain geometry, and the volume equations and mineral densities in (*88*). (n) Concentration of each element (He, U, Th and Sm) computed from the mass and the absolute amount of the measured isotopes (where ^{147}Sm is 0.15 of the total Sm reported here). In some cases Sm may not be measured, for example in minerals like zircon with negligible Sm. "n.m." indicates when Sm is not measured. (o) Uncertainties on U, Th, Sm, and He concentrations are reported at 2σ and include the propagated total analytical uncertainties (TAU). (p) eU is effective uranium concentration. Calculated as U + 0.238*Th + 0.0012*Sm after Appendix A of (*101*). (q) Uncertainty on eU is estimated at 15% of the eU value. (r) Uncorrected (U-Th)/He date is calculated iteratively using the ^{4}He production equation defined as equation 1 in (*102*) modified to include He produced from Sm decay and assuming secular equilibrium. (s) Uncertainty on the uncorrected (U-Th)/He date is reported at 2σ and includes the propagated total analytical uncertainties (TAU) on the U, Th, Sm and He measurements. (t) The combined alpha-ejection correction for the crystal calculated from the parent isotope-specific FT corrections, the proportion of U and Th contributing to ^{4}He production, and assuming homogeneous parent isotope distributions using equation A4 in (*101*). The parent isotope-specific alpha ejection-corrections were computed assuming the reported grain geometry in this table and the equations and alpha-stopping distances in (*88*). (u) The corrected (U-Th)/He date is calculated iteratively using the absolute values of He, U, Th and Sm, the isotope specific FT corrections, and equation 34 in (*88*) assuming secular equilibrium. (v) Uncertainty on the corrected (U-Th)/He date is reported at 2σ and includes the propagated total analytical uncertainties (TAU) on the U, Th, Sm and He measurements. Uncertainty propagation done using HeCalc (*103*). (x) Durango apatite fragments ran in conjunction with these analyses yield an unweighted mean and 2σ standard error of 30.8 ± 0.8 Ma (n=7). Fish Canyon Tuff zircon crystals ran in conjunction with these analyses yield an unweighted mean and 2σ standard error of 28.4 ± 1.0 Ma (n=7).

Table S7. Inverse Thermal History Model Input Information.
1. Simulations, Samples and Data Treatment
Samples: F1966
Note: Excluded F1966_z04 and F1966_z05 since these grains have high levels of radiation damage and ZRDAAM is less well-calibrated at high damage levels
Treatment: Samples were binned into groups with similar eU values as listed below, and the mean of each group was modeled
He dates (Ma): Mean uncorrected He date of each bin. a-ejection corrected in HeFTy using (*88*).
Error (Ma) applied in modeling: The 1σ sample standard deviation of each bin was applied if ≥15%. If <15%, then 15% was applied.
Rs (um): Mean equivalent spherical radius of each bin
U and Th (ppm): Mean U and Th for each bin
eU Bins:

Sample Name and aliquot	Rs (mm)	U (ppm)	±	Th (ppm)	±	Sm (ppm)	±	eU	±	Uncorr Date (Ma)	STD Date Uncertainty	15% Date Uncertainty
F1966_a06	67	0.29	0.07	0.42	0.04	33.6	1.8	0.43	0.07	110		
F1966_a05	87	0.31	0.07	0.49	0.04	38.5	1.1	0.47	0.07	220		
F1966_a04	57	0.28	0.06	0.57	0.10	53	3	0.48	0.07	280		
F1966_a03	58	0.20	0.06	1.09	0.10	47	3	0.51	0.08	160		
F1966_a02	60	0.36	0.09	0.82	0.08	70	3	0.64	0.10	240		
F1966_a01	42	1.23	0.15	2.9	0.3	217	12	2.2	0.3	188		
Bin 1 Averages	62	0.45	0.08	1.04	0.11	77	4	0.79	0.12	200	55	30
F1966_z02	182	102	5	97	2	n.m.	n.m.	125	19	600		
F1966_z01	186	113	4	120	4	n.m.	n.m.	140	20	564		
Bin 2 Averages	184	107	4	108.5	3.3	n.m.	n.m.	132	19	582	18	87
F1966_z03	38	480	30	430	30	n.m.	n.m.	590	90	330		
F1966_z06	51	466	17	770	20	n.m.	n.m.	600	100	340		
Bin 3 Averages	44	473	23	600	25	n.m.	n.m.	600	100	335	5	50

2. Additional Geologic Information Used to Impose t-T Constraints

Model A: Neoproterozoic Exhumation Hypothesis		
Time (Ma)	Temperature (°C)	Explanation
719.4–718.3	110–60	Test emplacement depth of 2–4 km (assuming 25°C/km geotherm and 10°C average surface temp)
515–500	20–0	Cambrian strata overlie basement rocks, indicating basement exposure by Cambrian time (e.g., *85*, *94*)
500–455	35–0	Early Paleozoic sedimentary xenolith suites in early Paleozoic kimberlite pipes indicate that strata of this age covered parts of the southwestern Slave craton (e.g., *85*, *94*)

Model B: Neoproterozoic Burial/Exhumation Hypothesis		
Time (Ma)	Temperature (°C)	Explanation
719.4–718.3	150–0	Test emplacement and subsequent sedimentary burial up to 6 km
719–514.5	150–0	Allows max Precambrian temperatures up to 150°C corresponding to sedimentary burial
515–500	20–0	Cambrian strata overlie basement rocks, indicating basement exposure by Cambrian time (e.g., *85*, *94*)

455–435	50–0	Increasing burial allowed in early Paleozoic time.
435–145	200–0	Exploration box to allow burial in Paleozoic-early Mesozoic time. Devonian xenoliths in the Jurassic Jericho kimberlite pipe indicate burial of the craton during the Devonian, which is corroborated by AHe data (*85*, *94*). Max temperature bound is based on the temperature sensitivity of ZHe.
145–140	20–0	Early Cretaceous sedimentary rocks unconformably overlie older units to the west of the craton, implying near-surface conditions in the Early Cretaceous (e.g., *85*, *94*)
140–75	200–0	Late Cretaceous sedimentary xenoliths occur in the 75–45 Ma Lac de Gras kimberlite field, indicating burial of the central craton by Cretaceous strata (summarized in *85*, *94*).
75–0	45–0	Early Cenozoic xenoliths in the 75–45 Ma Lac de Gras kimberlite field and associated thermal maturation studies suggest shallow burial depths (summarized in *85*, *94*).
0	20–0	At surface at present

500–455	35–0	Early Paleozoic sedimentary xenolith suites in early Paleozoic kimberlite pipes indicate that strata of this age covered parts of the southwestern Slave craton (e.g., *85*, *94*)
455–435	50–0	Increasing burial allowed in early Paleozoic time.
435–145	200–0	Exploration box to allow burial in Paleozoic-early Mesozoic time. Devonian xenoliths in the Jurassic Jericho kimberlite pipe indicate burial of the craton during the Devonian, which is corroborated by AHe data (*85*, *94*). Max temperature bound is based on the temperature sensitivity of ZHe.
145–140	20–0	Early Cretaceous sedimentary rocks unconformably overlie older units to the west of the craton, implying near-surface conditions in the Early Cretaceous (e.g., *85*, *94*)
140–75	200–0	Late Cretaceous sedimentary xenoliths occur in the 75–45 Ma Lac de Gras kimberlite field, indicating burial of the central craton by Cretaceous strata (summarized *85*, *94*).
75–0	45–0	Early Cenozoic xenoliths in the 75–45 Ma Lac de Gras kimberlite field and associated thermal maturation studies suggest shallow burial depths (summarized in *85*, *94*).
0	20–0	At surface at present

3. System- and model-specific parameters

He kinetic model: RDAAM for apatite (*83*); ZRDAAM for zircon (*84*)

Statistical fitting criteria: GOF values >0.5 for "good" fits. >0.05 for "acceptable" fits. The good-fits also must have a minimum GOF of 1/(N+1) where N is number of statistics used (*89*)

Modeling Code: HeFTy v1.9.3

Number of tT paths attempted: 25,000 per model

tT path characteristics: Monotonic variable heating or cooling, 8 segments between nodes, intermediate randomizer style, no maximum dT/dt imposed

REFERENCES AND NOTES

1. P. F. Hoffman, D. S. Abbot, Y. Ashkenazy, D. I. Benn, J. J. Brocks, P. A. Cohen, G. M. Cox, J. R. Creveling, Y. Donnadieu, D. H. Erwin, I. J. Fairchild, D. Ferreira, J. C. Goodman, G. P. Halverson, M. F. Jansen, G. Le Hir, G. D. Love, F. A. Macdonald, A. C. Maloof, C. A. Partin, G. Ramstein, B. E. J. Rose, C. V. Rose, P. M. Sadler, E. Tziperman, A. Voigt, S. G. Warren, Snowball Earth climate dynamics and Cryogenian geology-geobiology. *Sci. Adv.* **3**, e1600983 (2017).

2. M. T. Jones, D. A. Jerram, H. H. Svensen, C. Grove, The effects of large igneous provinces on the global carbon and sulphur cycles. *Palaeogeogr. Palaeoclimatol. Palaeoecol.* **441**, 4–21 (2015).

3. J. Kasbohm, B. Schoene, S. Burgess, Radiometric constraints on the timing, tempo, and effects of large igneous province emplacement, in *Large Igneous Provinces: A Driver of Global Environmental and Biotic Changes*, R. E. Ernst, A. J. Dickson, A. Bekker, Eds. (Geophysical Monograph 255, AGU and Wiley, ed. 1, 2021), pp. 27–82.

4. F. A. Macdonald, M. D. Schmitz, J. L. Crowley, C. F. Roots, D. S. Jones, A. C. Maloof, J. V. Strauss, P. A. Cohen, D. T. Johnston, D. P. Schrag, Calibrating the cryogenian. *Science* **327**, 1241–1243 (2010).

5. F. A. Macdonald, R. Wordsworth, Initiation of Snowball Earth with volcanic sulfur aerosol emissions. *Geophys. Res. Lett.* **44**, 1938–1946 (2017).

6. Y. Goddéris, Y. Donnadieu, A. Nédélec, B. Dupré, C. Dessert, A. Grard, G. Ramstein, L. M. François, The Sturtian “snowball” glaciation: Fire and ice. *Earth Planet. Sci. Lett.* **211**, 1–12 (2003).

7. G. M. Cox, G. P. Halverson, R. K. Stevenson, M. Vokaty, A. Poirier, M. Kunzmann, Z. X. Li, S. W. Denyszyn, J. V. Strauss, F. A. Macdonald, Continental flood basalt weathering as a trigger for Neoproterozoic Snowball Earth. *Earth Planet. Sci. Lett.* **446**, 89–99 (2016).

8. Y. Donnadieu, Y. Goddéris, G. Ramstein, A. Nédélec, J. Meert, A “snowball Earth” climate triggered by continental break-up through changes in runoff. *Nature* **428**, 303–306 (2004).

9. W. F. Fahrig, E. Irving, G. D. Jackson, Paleomagnetism of the Franklin diabases. *Can. J. Earth Sci.* **8**, 455–467 (1971).

10. S. W. Denyszyn, H. C. Halls, D. W. Davis, D. A. D. Evans, Paleomagnetism and U-Pb geochronology of Franklin dykes in high arctic Canada and Greenland: A revised age and paleomagnetic pole constraining block rotations in the Nares Strait region. *Can. J. Earth Sci.* **46**, 689–705 (2009).

11. L. M. Heaman, A. N. LeCheminant, R. H. Rainbird, Nature and timing of Franklin igneous events, Canada: Implications for a Late Proterozoic mantle plume and the break-up of Laurentia. *Earth Planet. Sci. Lett.* **109**, 117–131 (1992).

12. S. J. Pehrsson, K. L. Buchan, Borden dykes of Baffin Island, Northwest Territories: A Franklin U-Pb baddeleyite age and a paleomagnetic reinterpretation. *Can. J. Earth Sci.* **36**, 65–73 (1999).

13. S. W. Denyszyn, D. W. Davis, H. C. Halls, Paleomagnetism and U-Pb geochronology of the Clarence head dykes, Arctic Canada: Orthogonal emplacement of mafic dykes in a large igneous province. *Can. J. Earth Sci.* **46**, 155–167 (2009).

14. F. A. Macdonald, M. D. Schmitz, J. V. Strauss, G. P. Halverson, T. M. Gibson, A. Eyster, G. Cox, P. Mamrol, J. L. Crowley, Cryogenian of Yukon. *Precambrian Res.* **319**, 114–143 (2017).

15. S. E. Bryan, R. E. Ernst, Revised definition of large igneous provinces (LIPs). *Earth Sci. Rev.* **86**, 175–202 (2008).

16. J. M. Mattinson, Zircon U-Pb chemical abrasion ("CA-TIMS") method: Combined annealing and multi-step partial dissolution analysis for improved precision and accuracy of zircon ages. *Chem. Geol.* **220**, 47–66 (2005).

17. R. E. Ernst, D. P. G. Bond, S. Zhang, K. L. Buchan, S. E. Grasby, N. Youbi, H. El Bilali, A. Bekker, L. S. Doucet, Large igneous province record through time and implications for secular environmental changes and geological time-scale boundaries, in *Large Igneous Provinces: A Driver of Global Environmental and Biotic Changes*, R. E. Ernst, A. J. Dickson, A. Bekker, Eds. (Geophysical Monograph 255, AGU and Wiley, ed. 1, 2021), pp. 1–26.

18. A. N. LeCheminant, L. M. Heaman, Mackenzie igneous events, Canada: Middle Proterozoic hotspot magmatism associated with ocean opening. *Earth Planet. Sci. Lett.* **96**, 38–48 (1989).

19. W. Bleeker, M. A. Hamilton, U. Söderlund, "A Franklin age (716 Ma) for a large gabbro sill on Great Slave Lake, Northwest Territories, Canada" (Report No. A149, 2013); www.supercontinent.org.

20. R. E. Ernst, M. A. Hamilton, U. Söderlund, J. A. Hanes, D. P. Gladkochub, A. V. Okrugin, T. Kolotilina, A. S. Mekhonoshin, W. Bleeker, A. N. LeCheminant, K. L. Buchan, K. R. Chamberlain, A. N. Didenko, Long-lived connection between southern Siberia and northern Laurentia in the Proterozoic. *Nat. Geosci.* **9**, 464–469 (2016).

21. J. Ding, S. Zhang, D. A. D. Evans, T. Yang, H. Li, H. Wu, J. Chen, North China craton: The conjugate margin for northwestern Laurentia in Rodinia. *Geology* **49**, 773–778 (2021).

22. S. S. Harlan, L. Heaman, A. N. LeCheminant, W. R. Premo, Gunbarrel mafic magmatic event: A key 780 Ma time marker for Rodinia plate reconstructions. *Geology* **31**, 1053–1056 (2003).

23. W. Baragar, The Natkusiak Basalts, Victoria Island, District of Franklin (Paper 76-1A, Geological Survey of Canada, 1976), pp. 347–352.

24. J. Dostal, W. R. A. Baragar, C. Dupuy, Petrogenesis of the Natkusiak continental basalts, Victoria Island, Northwest Territories Canada. *Can. J. Earth Sci.* **23**, 622–632 (1986).

25. J. H. Bédard, B. Hayes, M. Hryciuk, C. Beard, N. Williamson, T. A. Dell'Oro, R. H. Rainbird, J. Prince, W. R. A. Baragar, P. Nabelek, D. Weis, B. Wing, J. S. Scoates, H. R. Naslund, B. Cousens, M.-C. Williamson, L. J. Hulbert, R. Montjoie, É. Girard, R. Ernst, C. J. Lissenberg, Geochemical database of Franklin sills, Natkusiak Basalts and Shaler Supergroup rocks, Victoria Island, Northwest Territories, and correlatives from Nunavut and the mainland (Open File Rep. 8009, Geological Survey of Canada, 2016; 1 .zip file, 10.4095/297842).

26. N. M. B. Williamson, L. Ootes, R. H. Rainbird, J. H. Bédard, B. Cousens, Initiation and early evolution of the Franklin magmatic event preserved in the 720 Ma Natkusiak Formation, Victoria Island Canadian Arctic. *Bull. Volcanol.* **78**, 1–19 (2016).

27. C. D. Beard, J. S. Scoates, D. Weis, J. H. Bédard, T. A. Dell'Oro, Geochemistry and origin of the Neoproterozoic Natkusiak flood basalts and related Franklin sills, Victoria Island Arctic Canada. *J. Petrol.* **58**, 2191–2220 (2018).

28. G. M. Young, The Amundsen Embayment, Northwest Territories; Relevance to the upper Proterozoic evolution of North America, in *Proterozoic Basins of Canada*, F. H. A. Campbell, Ed. (Paper 81-10, Geological Survey of Canada, 1981), pp. 203–218.

29. R. H. Rainbird, C. W. Jefferson, G. M. Young, The early Neoproterozoic sedimentary Succession B of northwestern Laurentia: Correlations and paleogeographic significance. *GSA Bull.* **108**, 454–470 (1996).

30. R. Thorsteinsson, E. T. Tozer, Banks, Victoria, and Stefansson Islands, Arctic Archipelago (Memoir 330, Geological Survey of Canada, 1962), pp. 85.

31. R. H. Rainbird, The sedimentary record of mantle plume uplift preceding eruption of the Neoproterozoic Natkusiak flood basalt. *J. Geol.* **101**, 305–318 (1993).

32. C. W. Jefferson, W. E. Nelson, R. V. Kirkham, J. H. Reedman, R. F. J. Scoates, Geology and copper occurrences of the Natkusiak basalts, Victoria Island, District of Franklin, in *Current Research, Part* A (Paper 85–1A, Geological Survey of Canada, 1985), pp. 203–214.

33. A. M. Durbano, B. R. Pratt, T. Hadlari, K. Dewing, Sedimentology of an early Cambrian tide-dominated embayment: Quyuk formation, Victoria Island Arctic Canada. *Sediment. Geol.* **320**, 1–18 (2015).

34. E. Burov, S. Cloetingh, Controls of mantle plumes and lithospheric folding on modes of intraplate continental tectonics: Differences and similarities. *Geophys. J. Int.* **178**, 1691–1722 (2009).

35. R. W. Carlson, Physical and chemical evidence on the cause and source characteristics of flood basalt volcanism. *Aust. J. Earth Sci.* **38**, 525–544 (1991).

36. T. A. Dell'Oro, "Sr-Nd-Hf-Pb isotope and trace element geochemistry of the Natkusiak Formation continental flood basalts of the Neoproterozoic Franklin large igneous province, Victoria Island, Canada," thesis, University of British Columbia, Vancouver, Canada (2012).

37. J. M. Mattinson, C. M. Graubard, D. L. Parkinson, W. C. McClelland, U-Pb reverse discordance in zircons: The role of fine-scale oscillatory zoning and sub-micron transport of Pb, in *Earth Processes:*

Reading the Isotopic Code, A. Basu, S. Hart, Eds. (Geophysical Monograph Series, AGU, 1996), vol. 95, pp. 355–370.

38. L. P. Black, P. D. Kinny, J. W. Sheraton, The difficulties of dating mafic dykes: An Antarctic example. *Contrib. Mineral. Petrol.* **109**, 183–194 (1991).

39. M. Ibanez-Mejia, G. E. Gehrels, J. Ruiz, J. D. Vervoort, M. E. Eddy, C. Li, Small-volume baddeleyite (ZrO_2) U-Pb geochronology and Lu-Hf isotope geochemistry by LA-ICP-MS. Techniques and applications. *Chem. Geol.* **384**, 149–167 (2014).

40. L. M. Heaman, A. N. LeCheminant, Anomalous U-Pb systematics in mantle-derived baddeleyite xenocrysts from Île Bizard: Evidence for high temperature radon diffusion? *Chem. Geol.* **172**, 77–93 (2000).

41. M. Rioux, S. Bowring, F. Dudás, R. Hanson, Characterizing the U-Pb systematics of baddeleyite through chemical abrasion: Application of multi-step digestion methods to baddeleyite geochronology. *Contrib. Mineral. Petrol.* **160**, 777–801 (2010).

42. P. S. Mustard, C. F. Roots, Rift-related volcanism, sedimentation, and tectonic setting of the Mount Harper Group, Ogilvie Mountains Yukon Territory. *Geol. Surv. Can. Bull.* **492**, 92 (1997).

43. J. A. Stevenson, J. L. Smellie, D. W. McGarvie, J. S. Gilbert, B. I. Cameron, Subglacial intermediate volcanism at Kerlingarfjöll, Iceland: Magma-water interactions beneath thick ice. *J. Volcanol. Geotherm. Res.* **185**, 337–351 (2009).

44. J. L. Smellie, Terrestrial subice volcanism: Landform morphology, sequence characteristics, environmental influences, and implications for candidate Mars examples. *GSA Spec. Pap.* **453**, 55–76 (2009).

45. Y. Donnadieu, F. Fluteau, G. Ramstein, C. Ritz, J. Besse, Is there a conflict between the Neoproterozoic glacial deposits and the snowball Earth interpretation: An improved understanding with numerical modeling. *Earth Planet. Sci. Lett.* **208**, 101–112 (2003).

46. D. Pollard, J. F. Kasting, Climate-ice sheet simulations of neoproterozoic glaciation before and after collapse to snowball Earth, in *The Extreme Proterozoic: Geology, Geochemistry, and Climate*, G. S. Jenkins, M. A. S. McMenamin, C. P. McKay, L. Sohl, Eds. (Geophysical Monograph Series, AGU, 2004), vol. 146, pp. 91–105.

47. S. MacLennan, Y. Park, N. Swanson-Hysell, A. Maloof, B. Schoene, M. Gebreslassie, E. Antilla, T. Tesema, M. Alene, B. Haileab, The arc of the Snowball: U-Pb dates constrain the Islay anomaly and the initiation of the Sturtian glaciation. *Geology* **46**, 539–542 (2018).

48. Z. Lan, M. H. Huyskens, K. Lu, X. H. Li, G. Zhang, D. Lu, Q. Z. Yin, Toward refining the onset age of Sturtian glaciation in South China. *Precambrian Res.* **338**, 105555 (2020).

49. G. M. Cox, J. V. Strauss, G. P. Halverson, M. D. Schmitz, W. C. McClelland, R. S. Stevenson, F. A. Macdonald, Kikiktat volcanics of Arctic Alaska-Melting of harzburgitic mantle associated with the Franklin large igneous province. *Lithosphere* **7**, 275–295 (2015).

50. J. C. G. Walker, P. B. Hays, J. F. Kasting, A negative feedback mechanism for the long-term stabilization of Earth's surface temperature. *J. Geophys. Res.* **86**, 9776–9782 (1981).

51. G. Li, J. Hartmann, L. A. Derry, A. J. West, C. F. You, X. Long, T. Zhan, L. Li, G. Li, W. Qiu, T. Li, L. Liu, Y. Chen, J. Ji, L. Zhao, J. Chen, Temperature dependence of basalt weathering. *Earth Planet. Sci. Lett.* **443**, 59–69 (2016).

52. C. Dessert, B. Dupré, J. Gaillardet, L. M. François, C. J. Allègre, Basalt weathering laws and the impact of basalt weathering on the global carbon cycle. *Chem. Geol.* **202**, 257–273 (2003).

53. C. Dessert, B. Dupré, L. M. François, J. Schott, J. Gaillardet, G. Chakrapani, S. Bajpai, Erosion of Deccan Traps determined by river geochemistry: Impact on the global climate and the $^{87}Sr/^{86}Sr$ ratio of seawater. *Earth Planet. Sci. Lett.* **188**, 459–474 (2001).

54. J. Hartmann, N. Moosdorf, R. Lauerwald, M. Hinderer, A. J. West, Global chemical weathering and associated P-release—The role of lithology, temperature and soil properties. *Chem. Geol.* **363**, 145–163 (2014).

55. Y. Park, N. L. Swanson-Hysell, L. E. Lisiecki, F. A. Macdonald, Evaluating the relationship between the area and latitude of large igneous provinces and Earth's long-term climate state, in *Large Igneous Provinces: A Driver of Global Environmental and Biotic Changes*, R. E. Ernst, A. J. Dickson, A. Bekker, Eds. (Geophysical Monograph 255, AGU and Wiley, ed. 1, 2021), pp. 153–168.

56. Y. Goddéris, G. Le Hir, M. Macouin, Y. Donnadieu, L. Hubert-Théou, G. Dera, M. Aretz, F. Fluteau, Z. X. Li, G. P. Halverson, Paleogeographic forcing of the strontium isotopic cycle in the Neoproterozoic. *Gondw. Res.* **42**, 151–162 (2017).

57. E. L. Taylor, T. N. Taylor, N. R. Cúneo, The present is not the key to the past: A polar forest from the permian of antarctica. *Science* **257**, 1675–1677 (1992).

58. R. H. Rainbird, R. E. Ernst, The sedimentary record of mantle-plume uplift. *Geol. Soc. Am. Spec. Paper* **352**, 227–245 (2001).

59. D. P. Schrag, R. A. Berner, P. F. Hoffman, G. P. Halverson, On the initiation of a snowball Earth. *Geochem. Geophys. Geosyst.* **3**, 1–21 (2002).

60. Y. Park, N. L. Swanson-Hysell, S. A. MacLennan, A. C. Maloof, M. Gebreslassie, M. M. Tremblay, B. Schoene, M. Alene, E. S. C. Anttila, T. Tesema, B. Haileab, The lead-up to the Sturtian Snowball Earth: Neoproterozoic chemostratigraphy time-calibrated by the Tambien Group of Ethiopia. *GSA Bull.* **132**, 1119–1149 (2019).

61. F. A. Macdonald, N. L. Swanson-Hysell, Y. Park, L. Lisiecki, O. Jagoutz, Arc-continent collisions in the tropics set Earth's climate state. *Science* **364**, 181–184 (2019).

62. Y. Park, P. Maffre, Y. Goddéris, F. A. MacDonald, E. S. C. Anttila, N. L. Swanson-Hysell, Emergence of the Southeast Asian islands as a driver for neogene cooling. *Proc. Natl. Acad. Sci. U.S.A.* **117**, 25319–25326 (2020).

63. Z. X. Li, D. A. D. Evans, G. P. Halverson, Neoproterozoic glaciations in a revised global palaeogeography from the breakup of Rodinia to the assembly of Gondwanaland. *Sediment. Geol.* **294**, 219–232 (2013).

64. N. R. McKenzie, B. K. Horton, S. E. Loomis, D. F. Stockli, N. J. Planavsky, C. T. A. Lee, Continental arc volcanism as the principal driver of icehouse-greenhouse variability. *Science* **352**, 444–447 (2016).

65. A. M. Friedrich, H. P. Bunge, S. M. Rieger, L. Colli, S. Ghelichkhan, R. Nerlich, Stratigraphic framework for the plume mode of mantle convection and the analysis of interregional unconformities on geological maps. *Gondw. Res.* **53**, 159–188 (2018).

66. E. Tziperman, I. Halevy, D. T. Johnston, A. H. Knoll, D. P. Schrag, Biologically induced initiation of neoproterozoic snowball-Earth events. *Proc. Natl. Acad. Sci. U.S.A.* **108**, 15091–15096 (2011).

67. C. E. Bucholz, C. J. Spencer, Strongly peraluminous granites across the archean-proterozoic transition. *J. Petrol.* **60**, 1299–1348 (2019).

68. M. J. Lewis, C. E. Bucholz, O. E. Jagoutz, Evidence for polybaric fractional crystallization in a continental arc: Hidden Lakes mafic complex, Sierra Nevada batholith California. *Contrib. Mineral. Petrol.* **176**, 1–27 (2021).

69. D. M. Johnson, P. R. Hooper, R. M. Conrey, XRF analysis of rocks and minerals for major and trace elements on a single low dilution li-tetraborate fused bead: JCPDS-International center for diffraction data. *Adv. X-Ray Anal.* **41**, 843–867 (1999).

70. R. M. Conrey, D. G. Bailey, J. W. Singer, L. Wagoner, B. Parfitt, J. Hay, O. Keh, Combined Use of Multiple Internal and External Standards in LA-ICPMS Analysis of Geologic Samples Using Lithium Borate Fused Glass, poster presented at the AGU Fall Meeting, San Francisco, CA, 9 December 2019.

71. P. Vermeesch, IsoplotR: A free and open toolbox for geochronology. *Geosci. Front.* **9**, 1479–1493 (2018).

72. K. L. Buchan, R. Ernst, Diabase dyke swarms of Nunavut, Northwest Territories and Yukon, Canada (Open File 7464, Geological Survey of Canada, 2013).

73. J. C. Harrison, M. R. St-Onge, O. V Petrov, S. I. Strelnikov, B. G. Lopatin, F. H. Wilson, S. Tella, D. Paul, T. Lynds, S. P. Shokalsky, C. K. Hults, S. Bergman, H. F. Jepsen, A. Solli, Geological map of the

Arctic / Carte géologique de l'Arctique (Map 2159A, Geological Survey of Canada, 2011), scale 1:5,000,000.

74. L. J. Hulbert, R. H. Rainbird, C. W. Jefferson, P. Friske, Map of Mafic and Ultramafic Bodies Related to the Franklin Magmatic Event, Minto Inlier, Victoria Island, NWT (Open File 4928, Geological Survey of Canada, 2005), scale 1:500,000.

75. J. Hiess, D. J. Condon, N. McLean, S. R. Noble, $^{238}U/^{235}U$ systematics in terrestrial uranium-bearing minerals. *Science* **335**, 1610–1614 (2012).

76. M. R. St-Onge, D. Scott, J, N. Rayner, M. Sanborn-Barrie, D. R. Skipton, B. M. Saumur, N. Wodicka, O. M. Weller, Archean and Paleoproterozoic cratonic rocks of Baffin Island, in *Geological Synthesis of Baffin Island (Nunavut) and the Labrador-Baffin Seaway*, L. T. Dafoe and N. Bingham-Koslowski, Eds. (Bulletin 608, Geological Survey of Canada, 2020), p. 29, 10.4095/321824.

77. D. J. Condon, B. Schoene, N. M. McLean, S. A. Bowring, R. R. Parrish, Metrology and traceability of U-Pb isotope dilution geochronology (EARTHTIME tracer calibration Part I). *Geochim. Cosmochim. Acta* **164**, 464–480 (2015).

78. T. E. Krogh, A low-contamination method for hydrothermal decomposition of zircon and extraction of U and Pb for isotopic age determinations. *Geochim. Cosmochim. Acta* **37**, 485–494 (1973).

79. M. D. Schmitz, B. Schoene, Derivation of isotope ratios, errors, and error correlations for U-Pb geochronology using ^{205}Pb-^{235}U-(^{233}U)-spiked isotope dilution thermal ionization mass spectrometric data. *Geochem. Geophys. Geosyst.* **8**, 1–20 (2007).

80. J. S. Stacey, J. D. Kramers, Approximation of terrestrial lead isotope evolution by a two-stage model. *Earth Planet. Sci. Lett.* **26**, 207–221 (1975).

81. C. Pin, J. S. Zalduegui, Sequential separation of light rare-earth elements, thorium and uranium by miniaturized extraction chromatography: Application to isotopic analyses of silicate rocks. *Anal. Chim. Acta* **339**, 79–89 (1997).

82. R. M. Flowers, P. K. Zeitler, M. Danišík, P. W. Reiners, C. Gautheron, R. A. Ketcham, J. R. Metcalf, D. F. Stockli, E. Enkelmann, R. W. Brown, (U-Th)/He chronology: Part 1. Data, uncertainty, and reporting. *GSA Bull.* (2022).

83. R. M. Flowers, R. A. Ketcham, D. L. Shuster, K. A. Farley, Apatite (U-Th)/He thermochronometry using a radiation damage accumulation and annealing model. *Geochim. Cosmochim. Acta* **73**, 2347–2365 (2009).

84. W. R. Guenthner, P. W. Reiners, R. A. Ketcham, L. Nasdala, G. Giester, Helium diffusion in natural zircon: Radiation damage, anisotropy, and the interpretation of zircon (U-TH)/He thermochronology. *Am. J. Sci.* **313**, 145–198 (2013).

85. A. K. Ault, R. M. Flowers, S. A. Bowring, Phanerozoic surface history of the Slave craton. *Tectonics* **32**, 1066–1083 (2013).

86. C. P. Sturrock, R. M. Flowers, F. A. Macdonald, The late great unconformity of the Central Canadian Shield. *Geochem. Geophys. Geosyst.* **22**, 1–22 (2021).

87. B. A. Peak, R. M. Flowers, F. A. Macdonald, J. M. Cottle, Zircon (U-Th)/He thermochronology reveals pre-great unconformity paleotopography in the Grand Canyon region USA.. *Geology* **49**, 1462–1466 (2021).

88. R. A. Ketcham, C. Gautheron, L. Tassan-Got, Accounting for long alpha-particle stopping distances in (U-Th-Sm)/He geochronology: Refinement of the baseline case. *Geochim. Cosmochim. Acta* **75**, 7779–7791 (2011).

89. R. A. Ketcham, Forward and inverse modeling of low-temperature thermochronometry data. *Rev. Mineral. Geochem.* **58**, 275–314 (2005).

90. R. M. Flowers, R. A. Ketcham, E. Enkelmann, C. Gautheron, P. W. Reiners, J. R. Metcalf, M. Danišík, D. F. Stockli, R. W. Brown, (U-Th)/He chronology: Part 2. Considerations for evaluating, integrating, and interpreting conventional individual aliquot data. *GSA Bull.* 10.1130/B36268.1 (2022).

91. U. Ginster, P. W. Reiners, L. Nasdala, C. Chanmuang N., Annealing kinetics of radiation damage in zircon. *Geochim. Cosmochim. Acta* **249**, 225–246 (2019).

92. W. R. Guenthner, Implementation of an Alpha Damage Annealing Model for Zircon (U-Th)/He Thermochronology With Comparison to a Zircon Fission Track Annealing Model. *Geochem. Geophys. Geosyst.* **22**, e2019GC008757 (2021).

93. R. A. Ketcham, HeFTy version 1.9.0 (2015).

94. P. F. Hoffman, Geology and tectonics, East Arm of Great Slave Lake, Northwest Territories (Map 1628A, Geological Survey of Canada, 1988), sheet 1 of 2, scale 1:250,000 and 1:500,000.

95. S. Polteau, A. Mazzini, O. Galland, S. Planke, A. Malthe-Sørenssen, Saucer-shaped intrusions: Occurrences, emplacement and implications. *Earth Planet. Sci. Lett.* **266**, 195–204 (2008).

96. A. K. Ault, R. M. Flowers, S. A. Bowring, Phanerozoic burial and unroofing history of the western Slave craton and Wopmay orogen from apatite (U-Th)/He thermochronometry. *Earth Planet. Sci. Lett.* **284**, 1–11 (2009).

97. A. K. Ault, R. M. Flowers, S. A. Bowring, Synchroneity of cratonic burial phases and gaps in the kimberlite record: Episodic magmatism or preservational bias? *Earth Planet. Sci. Lett.* **410**, 97–104 (2015).

98. W. F. McDonough, S.-s. Sun, The composition of the Earth. *Chem. Geol.* **120**, 223–253 (1995).

99. S.-s. Sun, W. F. McDonough, Chemical and isotopic systematics of oceanic basalts: Implications for mantle composition and processes. *Geol. Soc. Spec. Publ.* **42**, 313–345 (1989).

100. A. H. Jaffey, K. F. Flynn, L. E. Glendenin, W. C. Bentley, A. M. Essling, Precision measurement of half-lives and specific activities of ^{235}U and ^{238}U. *Phys. Rev. C* **4**, 1889–1906 (1971).

101. E. H. G. Cooperdock, R. A. Ketcham, D. F. Stockli, Resolving the effects of 2-D versus 3-D grain measurements on apatite (U-Th)/He age data and reproducibility. *Geochronology* **1**, 17–41 (2019).

102. R. A. Wolf, K. A. Farley, D. M. Kass, Modeling of the temperature sensitivity of the apatite (U-Th)/He thermochronometer. *Chem. Geol.* **148**, 105–114 (1998).

103. P. Martin, HeCalc (0.4.1). Zenodo (2022); 10.5281/zenodo.5672830.
